# (*E*)-2-Benzylidenecyclanones: Part XXI—Reaction of Cyclic Chalcone Analogs with Cellular Thiols: Comparison of Reactivity of (*E*)-2-Arylidene-1-Indanone with -1-Tetralone and -1-Benzosuberone Analogs in Thia-Michael Reactions

**DOI:** 10.3390/ijms262110573

**Published:** 2025-10-30

**Authors:** Csaba Kadlecsik, Gábor Bognár, Fatemeh Kenari, Zoltán Pintér, Júlio César de Oliveira Ribeiro, Mário G. Envall, Valter H. Carvalho-Silva, Hamilton B. Napolitano, Pál Perjési

**Affiliations:** 1 Institute of Pharmaceutical Chemistry, University of Pécs, H-7624 Pecs, Hungary; 2 Laboratory for Modeling of Physical and Chemical Transformations (MPhysChem), Theoretical and Structural Chemistry Group, Research and Graduate Center, Goiás State University, Anápolis 75132-903, Goiás, Brazil; 3 Chemistry Institute, Federal University of Goiás, Goiania 74690-900, Goiás, Brazil; 4 Group of Theoretical and Structural Chemistry of Anápolis (QTEA), State University of Goiás, Anápolis 75132-903, Goiás, Brazil

**Keywords:** chalcone, benzylideneindanones, benzylidenetetralones, benzylidenebenzosuberones, anticancer activity, glutathione, *N*-acetylcysteine, thia-Michael addition, molecular electrostatic, DFT calculations

## Abstract

In vitro cytotoxicity of three (*E*)-3-(4′-X-benzylidene)-1-indanones (**2a**-**c**) displayed lower cytotoxicity towards murine P388 and L1210 leukemic cells as well as human Molt 4/C8 and CEM T-lymphocytes than the respective six- (**3a**-**c**) and seven-membered (**4a**-**c**) analogs. To study whether thiol reactivity—as a possible basis of their mechanism of action—correlates with the observed cytotoxicities, kinetics of the non-enzyme catalyzed reactions with reduced glutathione (GSH) and *N*-acetylcysteine (NAC) of **2a**-**c** were investigated. Furthermore, it was also the aim of the work to compare the thiol reactivity of the open-chain chalcones (**1**) and their carbocyclic analogs (**2**-**4**) with different ring sizes (*n* = 5–7). The reactivity of the compounds and the stereochemical outcome of the reactions were evaluated using high-pressure liquid chromatography–mass spectrometry (HPLC-MS). Molecular modeling calculations were performed to rationalize the high initial rate and low conversion of the **2a** indanone in comparison with those of the carbocyclic analog tetralone (**3a**) and benzosuberone (**4a**). Thiol reactivity and cancer cell cytotoxicity showed a dependence on both the ring size and the nature of aromatic substituents.

## 1. Introduction

Chalcones (**1**) are natural compounds, most often found in higher plants, especially in members of the *Leguminosae*, *Asteraceae*, and *Moraceae* families [1,2]. These plants are commonly used in traditional medicine, and as a result, the anti-inflammatory, antimicrobial, antifungal, antiviral, antidiabetic, and chemopreventive effects of chalcones have been extensively studied in many laboratories. Recognizing the diverse biological effects of natural chalcones, synthesis of a large number of synthetic chalcones and chalcone analogs, as well as investigation of their biological effects, have begun and are ongoing [2,3,4,5,6,7,8,9,10]. Among the recognized mechanisms of action of the compounds, NF-κB pathway inhibition (anti-inflammatory effect) [11,12], activation of the Keap1/Nrf2/ARE pathway (antitumor/cytoprotective effect) [12,13,14], and inhibition of protein kinases (antitumor effect) [15,16]) have been considered (at least in part) to be a consequence of their Michael-type reactivity toward the reactive cysteine residues of proteins.

**Figure 1:**
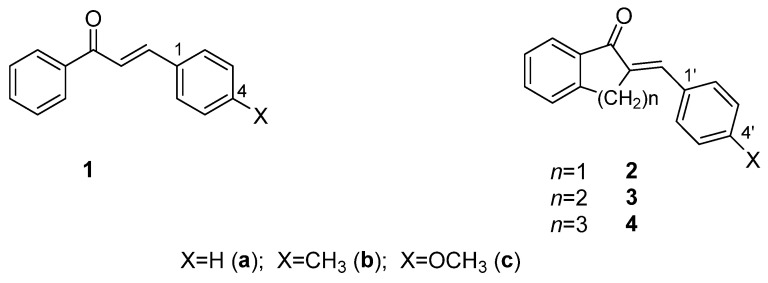
Structure of chalcones (**1**) and their cyclic analogs (**2**-**4**).

Since the different three-dimensional structures of chalcones exhibit different physicochemical properties, different reactivities, and noncovalent interactions, the synthesis and investigation of conformationally restricted chalcone derivatives (such as cyclic chalcone analogs) are useful molecular tools for both chemical and biological comparative studies. 2-Arylidene-benzocyclan-1-ones (**2**-**4**) can be considered analogs of chalcones (**1**) restricted in conformational flexibility [17] (Figure 1). Among them, for example, several indanone derivatives (**2**) have been published, which have an effective antitumor (tubulin-active) effect [18,19,20,21,22,23].

These conformationally constrained cyclic chalcone analogs have a three-dimensional structure not found in conformational equilibrium mixtures of open-chain analogs [24]. Therefore, these compounds—and their analogs with different ring sizes—are useful “molecular tools” for investigating how the size and structure of the ring influence the three-dimensional shape of the chalcone structural unit. Furthermore, various three-dimensional structures result in different degrees of conjugation, which can affect the covalent and noncovalent interactions of the compounds.

Earlier, we investigated the cytotoxicity of systematically substituted chalcones (**1**) and cyclic chalcone analogs (**2**-**4**) against murine P388 and L1210 leukemic cells, as well as human Molt 4/C8 and CEM T-lymphocytes [25,26]. Selected data are presented in Table S1. Furthermore, we have investigated the thiol reactivity of **1b**, **c**, **3b**, **c**, and **4b**, **c** to establish a correlation between thiol reactivity and the cytotoxicity of the derivatives against different cancer cell lines [27,28,29].

The present work aimed to compare thiol reactivity of selected open-chain chalcones (**1a**-**c**) with their five (**2a**-**c**), six- (**3a**-**c**), and seven-membered (**4a**-**c**) carbocyclic analogs, and to seek a correlation between the cytotoxicity and thiol reactivity of the related compounds with the same substitution pattern but different ring size. In addition, it was also the aim of the work to compare the thiol reactivity of the open-chain chalcones (**1**) and their carbocyclic analogs (**2**-**4**) with different ring sizes (*n* = 5–7) with the deprotonated (RS^−^) and the neutral (RSH) thiols.

Similarly to our previous studies [27,28], for the sake of comparisons, reduced glutathione (GSH) and *N*-acetylcysteine (NAC) were used as model thiol compounds. The reactions were run under three different pH conditions (pH 8.0, pH 6.3, and pH 3.2). The basic pH was selected to mimic the ionization state of GSH at the active site of the glutathione-S-transferase (GST) enzymes [29]. The slightly acidic pH resembles that of the cancer cells [30]. Under the strongly acidic pH (pH 3.2), both thiols exist exclusively in protonated (neutral) form [31]. Accordingly, under such conditions, reactivity of the neutral thiol nucleophiles can be investigated.

**Figure 2:**
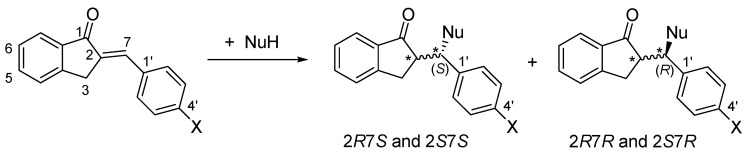
A simplified reaction scheme of the addition of GSH and NAC (NuH) onto the cyclic chalcone analogs. The * indicates chiral carbon atom.

As a result of the addition reactions, two new chiral centers are formed. Considering the inherent chirality of GSH (2*S*, 5*R*) and NAC (*R*), formation of four diastereomeric adducts was expected (Figure 2). Besides monitoring the progress of the reactions (as indicated by the reduction in the peak areas of the chalcones), the HPLC-UV method also provided information on the diastereomeric distribution of the isomeric adducts under different experimental conditions.

The incubation mixtures were thermostated at 37 °C. HPLC-UV analyses were performed over 315 min. To gain a better understanding of the kinetic profiles of the reactions, molecular modeling calculations were conducted. These analyses used methanethiol (**CH_3_SH**) and its deprotonated form (**CH_3_S^−^**) as model thiols.

## 2. Results

### 2.1. Reactions Under Slightly Basic (pH 8.0) Conditions

Initially, we investigated the reactions of the three chalcone analogs (**2a**-**c**) under basic conditions (pH 8.0). Taking into consideration the pK_a_ values of GSH (pK_a_ 8.83) and NAC (pK_a_ 9.52) [31], 12.9% of the GSH and 2.9% of the NAC molecules exist in the more reactive thiolate form. As expected, GSH showed higher reactivity toward each chalcone analog than NAC. By the end of the investigated period (315 min), the area of the HPLC-UV peak of the parent compounds **2a**, **2b**, and **2c** had reduced to 54.5%, 63.9%, and 84.9% of their initial values, respectively (Table 1). While the compounds were incubated with the less reactive NAC, the respective figures were 59.4%, 71.3%, and 87.5% (Table 2). In the control experiments (incubations without the thiols) no reduction in the initial chromatographic peak of **2a**-**c** was observed. (Similar results were obtained in the pH 6.3 and pH 3.2 control incubations.)

**Figure 3:**
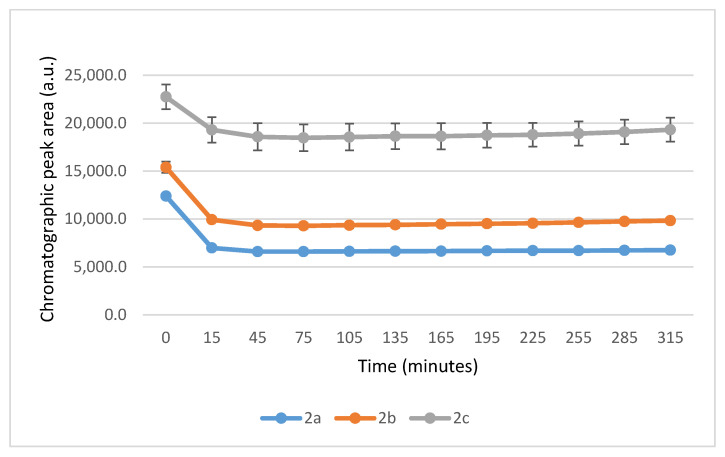
Change in the HPLC-UV chromatographic peak area of (*E*)-2-arylidene-1-indanones **2a**-**c** as a function of time (min) in the **2a**-**c**/GSH incubations at pH 8.0. Each data point represents the average of two independent measurements. Error bars indicate the deviation, calculated as half the difference between two independent measurements. Due to high reproducibility, error bars may not be visually apparent at some points.

**Figure 4:**
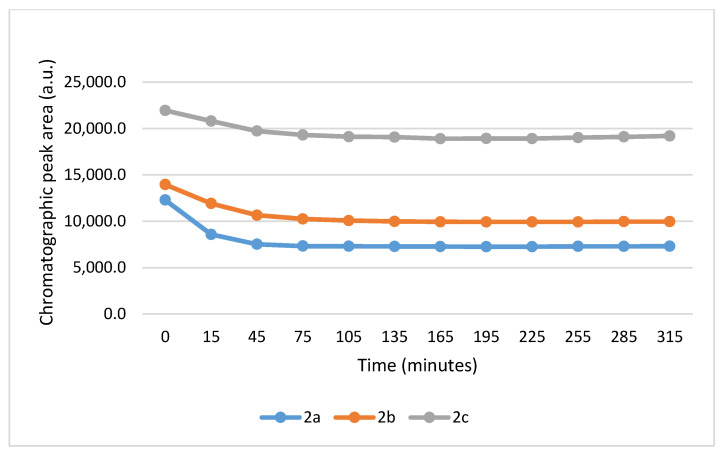
Change in the HPLC-UV chromatographic peak area of (*E*)-2-arylidene-1-indanones **2a**-**c** as a function of time (min) in the **2a**-**c**/NAC incubations at pH 8.0. Each data point represents the average of two independent measurements. Error bars indicate the deviation, calculated as half the difference between two independent measurements. Due to high reproducibility, error bars may not be visually apparent at some points.

It is worth noting that the reactions are very fast. The reactions with GSH reached equilibrium by the 45 min timepoint (Figure 3) while those with NAC reached equilibrium by the 75 min timepoint (Figure 4).

**Figure 5:**
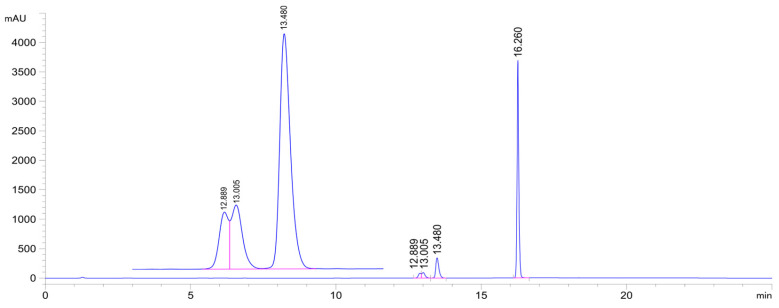
HPLC-UV chromatogram of the **2c/GSH** incubate (pH 6.3; 315 min sample). (**2c**: t_r_16.26 min, **2c-GSH-1a** conjugate: t_r_12.89 min, **2c-GSH-1b** conjugate: t_r_13.01 min, **2c-GSH-2** conjugate: t_r_13.48 min.).

Addition of both thiols results in formation of four diastereomers (Figure 2). Similarly to previous studies [28], the HPLC-UV chromatograms exhibit two distinct groups of peaks. In the case of the GSH adducts, the adducts with shorter retention times (**GSH-1**) give two partly separated peaks (**GSH-1a** and **GSH-1b**), and those with the higher retention times (**GSH-2**) provide only one asymmetric one (Figure 5, Figures S1 and S2).

In the case of the NAC-adducts, the chromatograms show the opposite pattern: the adducts with the shorter retention times (**NAC-1**) give one peak, and those with the higher retention times (**NAC-2**) give two partially separated peaks (**NAC-2a** and **NAC-2b**) (Figure 6, Figures S3 and S4). The structure of the parent chalcone analogs (**2a**-**c**) (Figures S5–S13), the formed GSH-adducts (Figures S14–S22), and NAC-adducts (Figures S23–S31) was supported by HPLC-MS.

Stereochemistry of the particular diastereomers appearing in the chromatograms is not known. While investigating the similar reactions of the open-chain **1a** (present work), as well as **1b** and **1c** [27] (where only two diastereomeric adducts can be formed) with NAC, the two diastereomeric adducts appeared in the HPLC-UV chromatograms as two partially separated peaks. The ratio of the two peaks in the **1b**/NAC incubation changed as a function of the incubation time. The initially formed more polar adduct (**NAC-1**) underwent isomerization to the least polar thermodynamic product (**NAC-2**). The observed transformation can be rationalized by the retro-Michael reaction of the kinetic product. Accordingly, the two diastereomers differ in the configuration of the newly formed chiral center of the adducts [27].

In the present experiments, the initial (15 min) ratio (between 2.0 and 2.2) of A(GSH-2)/A(GSH-1) decreased (to between 1.5 and 1.6) and remained unchanged after the 45 min timepoint (Table 3). The initial A(NAC-2)/A(NAC-1) ratios were found to be much higher (between 3.1 and 3.9) at the 15 min timepoint and continuously decreased and reached similar 1.5–1.6 (final) values at the 195 min timepoint (Table 4). These observations can also be rationalized by the fast conversion of the kinetic to the thermodynamic products.

Considering the reversibility of the addition reactions, formation of the respective (*Z*)-isomers cannot be excluded. To characterize the structure and chromatographic behavior of the (*Z*)-isomers, light-initiated isomerization of **2a**-**c** was performed [32]. The structure of the (*Z*)-isomers of **2a**-**c** was identified by HPLC-MS (Figures S5–S13). Under the conditions of the used HPLC-UV method, however, the (*E*) (*Z*) pairs of the investigated cyclic chalcone analogs did not separate. To investigate the possible formation of the respective (*Z*)-isomers, the incubations were also analyzed by the previously published HPLC-UV method, which can separate the two geometric isomers [32]. HPLC peaks corresponding to the respective (*Z*) isomers could not be detected in any incubate (Table 1 and Table 2).

### 2.2. Reactions Under Slightly Acidic (pH 6.3) Conditions

**Figure 7:**
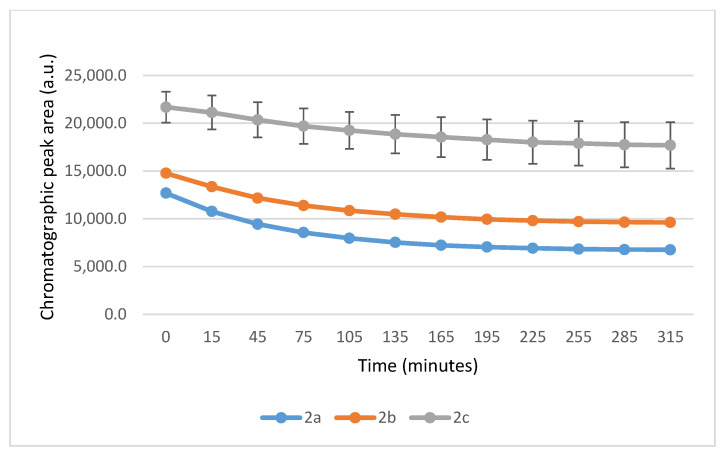
Change in the HPLC-UV chromatographic peak area of (*E*)-2-arylidene-1-indanones **2a**-**c** as a function of time (min) in the **2a**-**c**/GSH incubations at pH 6.3. Each data point represents the average of two independent measurements. Error bars indicate the deviation, calculated as half the difference between two independent measurements (*n* = 2). Due to high reproducibility, error bars may not be visually apparent at some points.

**Figure 8:**
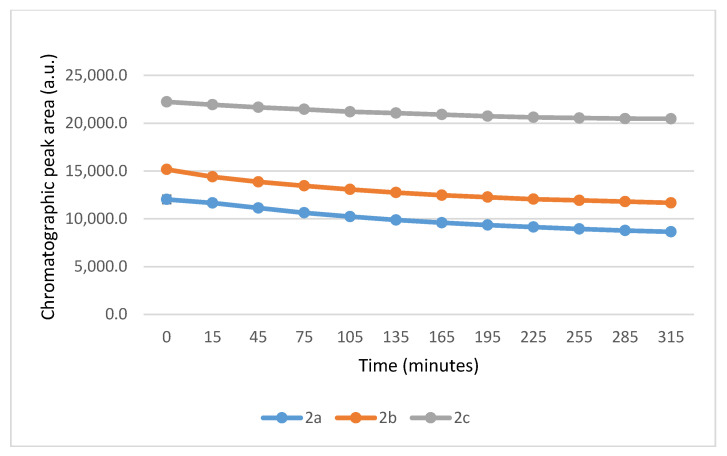
Change in the HPLC-UV chromatographic peak area of (*E*)-2-arylidene-1-indanones **2a**-**c** as a function of time (min) in the **2a**-**c**/NAC incubations at pH 6.3. Each data point represents the average of two independent measurements. Error bars indicate the deviation, calculated as half the difference between two independent measurements. Due to high reproducibility, error bars may not be visually apparent at some points.

Under these slightly acidic conditions, about 0.3% of the GSH and 0.06% of the NAC molecules exist in the more reactive thiolate form. Accordingly, the progress of the reactions was slower than that observed at pH 8.0. In the GSH incubations, the peak area of the parent compounds **2a**, **2b**, and **2c** was reduced by the 315 min timepoint to 53.3%, 65.1%, and 81.6% of the initial value, respectively (Table 1). The corresponding figures in the **2a**-**c**/NAC incubations (at the 315 min timepoint) were 71.9%, 77.0%, and 92.1%, respectively (Table 2). The progress of the reaction also showed characteristic differences between GSH and NAC incubations. While the GSH incubations reached equilibrium by the 195 min timepoints, the NAC incubations reached equilibrium only towards the end of the incubation period (315 min) (Figure 7 and Figure 8).

In comparison to the pH 8.0 values, the A(GSH-2)/A(GSH-1) ratios were found to be somewhat different. The initially high ratios (between 2.7 and 3.4) slowly but continuously reduced and stabilized at about 2 (1.95–2.0) by the end of the incubations (Table 3). On the contrary, the initial A(NAC-2)/A(NAC-1) ratios (between 0.9 and 1.4) were slightly but continuously increased and stabilized around 1.4 (between 1.3 and 1.5) by the end of the incubations (Table 4). No HPLC peaks corresponding to the respective (*Z*) isomers could be detected in the incubates (Table 1 and Table 2).

### 2.3. Reactions Under Acidic (pH 3.2) Conditions

Under the acidic conditions, the reactions of the protonated thiol function of the two model compounds were investigated. As expected, the reactivity of thiols under such conditions became even slower. In the GSH incubations, the peak areas of **2a**, **2b**, and **2c** were reduced to 82.2%, 78.4%, and 92.4% of their initial values, respectively, by the end of the incubation (Table 1). Under the same conditions, the initial peak area of **2a**, **2b**, and **2c** was reduced to 67.2%, 88.9%, and 94.7%, respectively (Table 2). The curves showing the progress of the reactions (decrease in the amount of starting chalcone analogs) are linear with a low slope. The unexpectedly high (32.8%) reduction in the initial peak area of **2a** in the NAC incubation can be rationalized by several peaks in the HPLC-UV chromatogram, in addition to those of the NAC-conjugates (Figure S32). Similar unexpectedly high decreases in the seven-membered **4b** and **4c** with NAC under pH 3.2 conditions were observed before [28]. Like the results obtained under basic (pH 8.0) and slightly acidic (pH 6.3) conditions, the order of reactivity of the three chalcone analogs with different substitution patterns with both thiols was **2a** > **2b** > **2c** (Table 1 and Table 2).

The initial A(GSH-2)/A(GSH-1) ratio of compounds **2a**, **2b**, and **2c** (2.45, 2.77, and 2.36, respectively) continuously increased over the time of incubation. By the end of the incubations, the ratios increased to 2.90 (**2a**), 3.71 (**2b**), and 4.25 (**2c**) (Table 3). On the contrary, the initial (15 min) A(NAC-2)/A(NAC-1) ratios were unexpectedly low (between 0.32 and 0.39) and only slightly changed (between 0.38 and 0.50) over the time of incubations. It is worth noting that the ratios observed under these conditions are opposite to those observed under pH 6.3 and pH 8.0 conditions (Table 4).

### 2.4. Molecular Modeling Analysis

Table 5 presents the calculated values for the molecular properties of **1a**, **2a**, **3a**, **4a**, methanethiol (**CH_3_SH**), and the deprotonated form of methanethiol (**CH_3_S^−^**). The HOMO energy of **CH_3_SH** is more negative than that of the **CH_3_S^−^**. The LUMO energy of **1a** and the carbocyclic chalcone analogs (**3a** and **4a**) increases in parallel with the ring size (*n*); (**1a**: *n* = 0). The LUMO energies of **2a** are similar to those of **4a** (Table 5).

**Figure 9:**
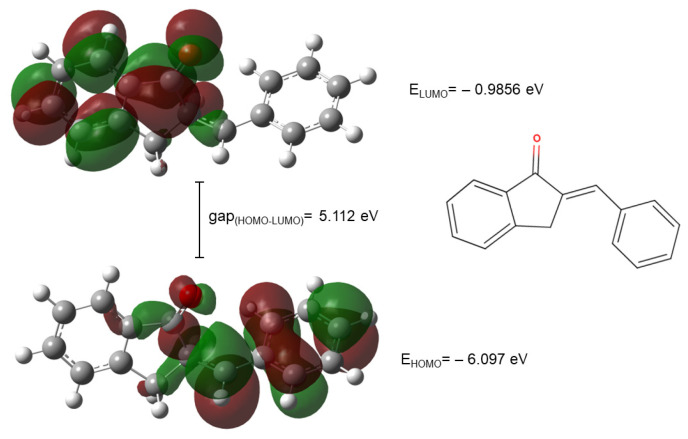
The HOMO and LUMO plots of compound **2a**. (The green and red lobes correspond to the positive and negative phases of the molecular orbitals, respectively).

The HOMO and LUMO plots of compounds **2a** are shown in Figure 9.

## 3. Discussion

(*E*)-2-Arylidene-1-indanones (**2**) are a family of compounds with a wide range of advantageous biological activities [18,19,20,21,22,23]. The mechanism of action of the compounds at the molecular level is not investigated in most of the reported cases. Being α,β-unsaturated ketones, the Michael-type reaction of compounds **2a**-**c** with reactive cellular thiols is one of the possible molecular mechanisms of the biologically active derivatives [12,13,14,15].

A comparison of the cancer cell cytotoxic IC_50_ values for approximately 80 derivatives revealed that the (*E*)-2-arylidene-1-indanones (**2**) were the least toxic cyclic chalcone analogs [25,26]. On the other hand, the average IC_50_ data for the six-membered (**3**) and seven-membered (**4**) analogs showed similar values (Table 6).

Thiol-reactivity, a possible (at least partly) basis of the cytotoxic effect of the compounds, has been investigated among the series **1**, **3**, and **4** [27,28]. Here, we report on the thiol reactivity of **2a**-**c**, investigated under the same conditions as before.

The pH-dependence of thiol-reactivity of **2a**-**c** showed a similar pattern to that previously observed: the higher the concentration of the more reactive thiolate, the higher the reactivity. The effect of substituents is also the same as it was before: the 4′-methoxy-substituted derivative (**2c**) showed lower reactivity than the 4′-methyl one (**2b**). In the present work, the reactivity of the non-substituted **2a** was also investigated.

^13^C NMR shifts—indicating the electron density around the particular nucleus [33]—of the C7 atom of **2a** (133.9 ppm), **2b** (133.9 ppm), and **2c** (133.7 ppm) were reported to be very similar [34]. According to the generally accepted mechanism of addition of the thiolate nucleophiles to polar carbon–carbon double bonds, derivatives with the most electron-deficient beta-carbon atoms show the highest reactivity [35]. Although the slight difference between the chemical shifts of **2b** and **2c** is in accordance with the somewhat lower reactivity of **2c**, the similar δ ^13^C(7) values of **2a** and **2b**, however, do not give a rationale for the higher reactivity of the unsubstituted **2a** (Table 1 and Table 2).

**Figure 10:**
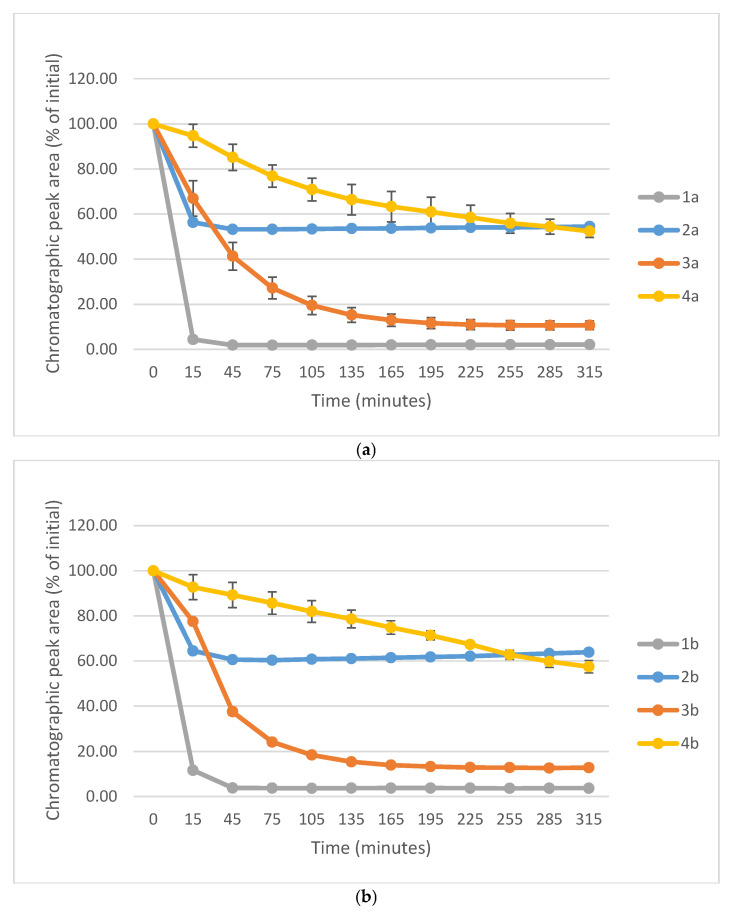

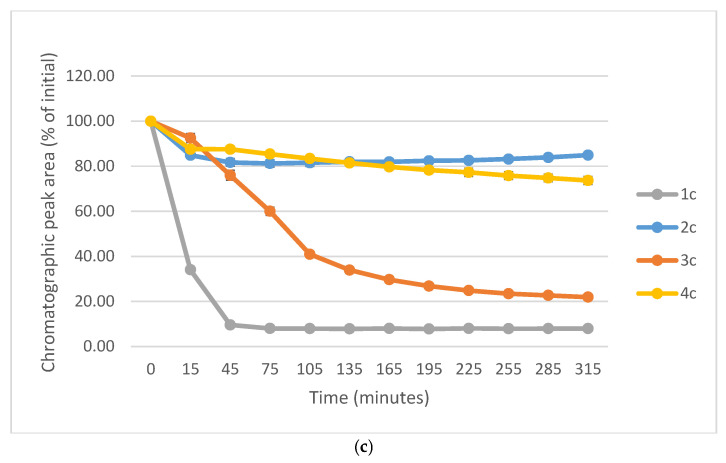
(**a**) Comparison of the change in HPLC-UV chromatographic peak areas of **1a** and its cyclic chalcone analogs (**2a**, **3a**, and **4a**) as a function of time (min) in the GSH incubations, pH 8.0. Each data point represents the average of two independent measurements. Error bars indicate the deviation, calculated as half the difference between two independent measurements. Due to high reproducibility, error bars may not be visually apparent at some points. (**b**) Comparison of the change in HPLC-UV chromatographic peak areas of **2a** and its cyclic chalcone analogs (**2b**, **3b**, and **4b**) as a function of time (min) in the GSH incubations, pH 8.0. Each data point represents the average of two independent measurements. Error bars indicate the deviation, calculated as half the difference between two independent measurements. Due to high reproducibility, error bars may not be visually apparent at some points. (**c**) Comparison of the change in HPLC-UV chromatographic peak areas of **1c** and its cyclic chalcone analogs (**2c**, **3c**, and **4c**) as a function of time (min) in the GSH incubations, pH 8.0. Each data point represents the average of two independent measurements. Error bars indicate the deviation, calculated as half the difference between two independent measurements. Due to high reproducibility, error bars may not be visually apparent at some points.

Figure 10a–c show a comparison of the change in HPLC-UV chromatographic peak areas of selected chalcones (**1a**-**c**) and their carbocyclic chalcone analogs (**2**a-**c**; **3a**-**c**; and **4a**-**c**) as a function of time (min) in the GSH incubations at pH 8.0. As shown, the open-chain chalcones (**1a**-**c**) showed the highest reactivity towards GSH. On the other hand, the **2a**-**c** and **4a**-**c** derivatives showed the lowest one. In each series reactivity of the 4′-methoxy-substituted derivative (**1c**-**4c**) showed lower reactivity than the respective methyl-substituted ones (**1b**-**4b**). Furthermore, the unsubstituted derivatives (**1a**-**4a**) showed the highest tiol-reactivity in each series. Similar observations were made while investigating the reactivity of the compounds with NAC under the same conditions (Figure S33). These observations support the theory of Allen and Hamphlett [36], considering the electron-donating capacity of the 4′-aryl substituents as a main driving force of the reverse process.

Addition of thiolates to polarized carbon–carbon double bonds is an orbital-controlled process [37,38,39,40]. This type of interaction between a vacant orbital and an electron pair requires conditions with a sufficiently small energy separation and sufficient overlap between the orbitals. A comparison of HOMO-LUMO interactions among various compounds reveals that a higher HOMO and lower LUMO result in a smaller energy separation, making them a more favorable combination for electron acceptor and donor roles in charge or electron transfer.

To investigate the HOMO-LUMO overlapping of **2a**, and compare it with the respective open-chain chalcone (**1a**) and the carbocyclic chalcone analogs **3a** and **4a**, molecular modeling calculations were performed using methanethiol (**CH_3_SH**) methylthiolate (**CH_3_S^−^**) anion as a model thiol. The HOMO-LUMO gap energies are summarized in Table 7.

**Figure 11:**
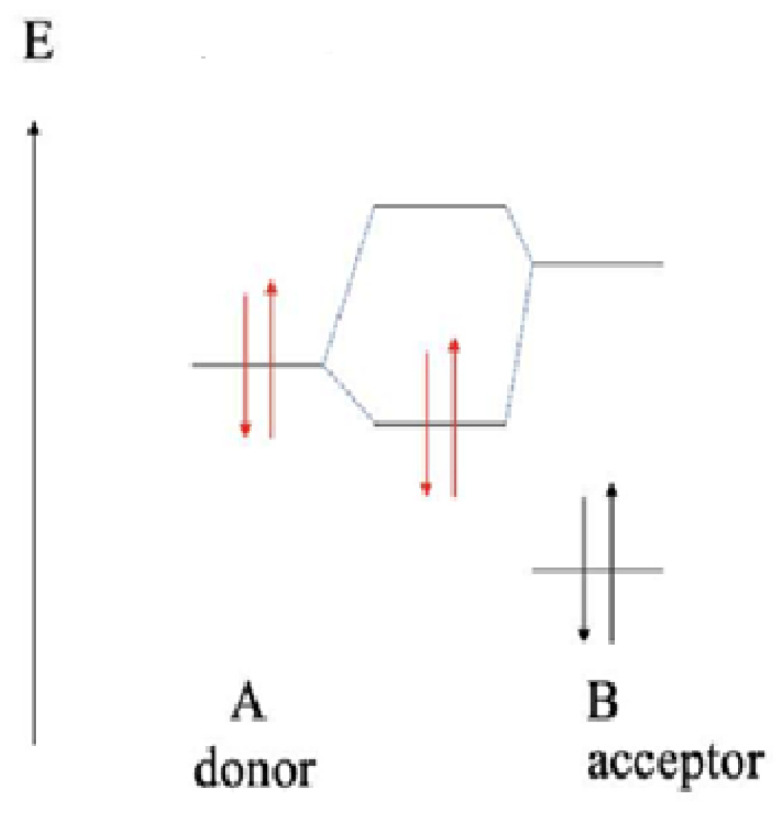
Simplified energy (*E*) scheme of the HOMO and LUMO molecular orbital energies of the donor (A) thiol/thiolate (HOMO) and the acceptor (B) chalcones (LUMO). The red and black arrows represent electrons with opposite spins in the molecular orbitals of the donor and acceptor, respectively.

According to the theory of orbital-controlled process, the energy gaps are lower between the *E*_LUMO_ of chalcones and *E*_HOMO_ of the more reactive thiolate (**CH_3_S^−^**). Furthermore, the HOMO energies of the donor thiol/thiolate are lower than the LUMO energies of acceptor chalcones (Table 7). Such interactions result in formation of a polar dative bond, which is polarized towards the sulfur atom (Figure 11).

The order of Δ*E*_HOMO-LUMO_ (**CH_3_S^−^**) and the Δ*E*_HOMO-LUMO_ (**CH_3_SH**) values **1a** < **3a** < **4a** is the same as the observed thiol-reactivity of the respective compounds—such a finding provides further proof of the orbital-controlled nature of the thia-Michael reactions. The value of **2a**, however, does not fit these tendencies.

**Figure 12:**
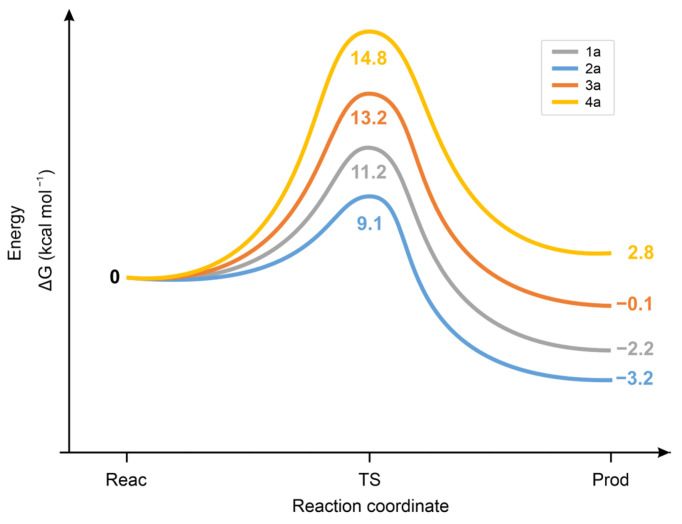
Standard free energy change (ΔG) for the transition state (TS) formation of the forward and the backward reactions of chalcone (**1a**) and its carbocyclic analogs (**2a**-**4a**) with methylthiolate (**CH_3_S^−^**) anion.

Calculation of the standard Δ*G* values of the chalcone. **CH_3_S^−^** reactions showed the **2a** < **1a** < **3a** < **4a** order for the forward and the **2a** < **3a** = **1a** < **4a** for the backward reaction (Figure 12). The unexpectedly low Δ*G* value of the **2a-CH_3_S**^−^ transition state is likely due to the high increase in conformational freedom accompanied by its formation from the rigid, strongly conjugated (*E*)-2-benzylidene-1-indanone (**2a**) [41].

To further evaluate the thiol-reactivity of chalcone derivatives, kinetic measurements were performed using methylthiolate (**CH_3_S^−^**) as the nucleophile at varying concentrations. The half-life values obtained for the reactions of compounds **1a**–**4a** with **CH_3_S^−^** are presented in Table 8. These data provide insights into the relative reactivity of the different structural analogs under nucleophilic attack, helping to clarify the influence of ring size and electronic effects on the reaction rate.

The results in Table 8 show that compound **2a** consistently exhibits the shortest half-lives across all **CH_3_S^−^** concentrations, indicating the highest reactivity towards the thiolate nucleophile. In contrast, compound **4a** shows the most prolonged half-lives, suggesting significantly lower reactivity. This trend follows the order **2a** > **1a** > **3a** > **4a** in terms of reactivity. Additionally, as the concentration of **CH_3_S^−^** increases, the half-lives decrease for all compounds, reflecting the expected behavior of second-order nucleophilic addition reactions. These findings support the theory that orbital interactions—particularly the alignment and energy of LUMO orbitals—play a critical role in determining the thiol-reactivity of these chalcone analogs [42].

The reaction of the compounds under acid conditions showed different features compared with those obtained under pH 8.0 and pH 6.3 conditions. Reactivity of the neutral thiol function is lower than that of its deprotonated counterparts [40]. Accordingly, the conversions are the lowest in these experiments (Table 1 and Table 2). Furthermore, under such conditions, several other (oxidative) products than the GSH/NAC adducts were detected in the HPLC chromatograms. Accordingly, the thiol-reactivity of compounds **2** cannot be compared with the results obtained in the pH 6.3 and pH 8.0 incubations.

**Figure 13:**
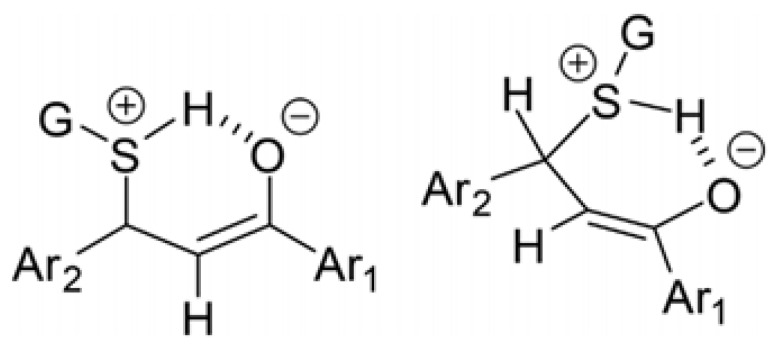
Possible cyclic intermediate of the addition of protonated glutathione (GSH) onto the enone carbon–carbon double bond [27].

Regardless of the nature of the thiol, the degree of diastereoselectivity of the addition process increases in parallel with the decrease in pH (Table 3 and Table 4). Addition of the neutral thiols to enones results in a zwitterion intermediate. The formed zwitterion can be stabilized by intermolecular hydrogen bonding, resulting in a conformationally preferred six-membered cyclic intermediate [27] (Figure 13).

Due to the high basicity of the enolate intermediate, intramolecular proton transfer can occur, leading to the formation of the final product. Since formation of the cyclic intermediate is a conformation-governed process, the preferred conformation with the pseudoequatorial position of the side-chain aromatic ring determines the *Re*-side attack of the nucleophiles and the stereochemistry of ketonization of the enolate moiety. This effect is most pronounced in the reaction of the weaker acidic NAC under pH 3.2 conditions. Under these conditions, the relative amount of the more polar **NAC-1** adducts is much (2–3 times) higher than the **NAC-2** adducts, which are the dominant products under the less acidic (pH 6.3 and pH 8.0) conditions (Table 2).

Changing the relative amount of the less polar adducts (**GSH-2** and **NAC-2**) as a function of pH shows opposite tendencies. In the case of the NAC-adducts, the lower the pH, the lower the relative amount of the less polar (**NAC-2**) adducts. In the case of GSH, an opposite tendency could be observed. This observation highlights the effect of the different substituents (G) on the protonated sulfur atom that forms the cyclic intermediate (Figure 13). Similarly, conformation-derived stereochemistry was observed in the formation of 2-aryl-substituted bicyclyc 1,3-oxazines formed in the cyclization reaction of alicyclic 1,3-aminoalkohols with aromatic aldehydes [43].

**Figure 14:**
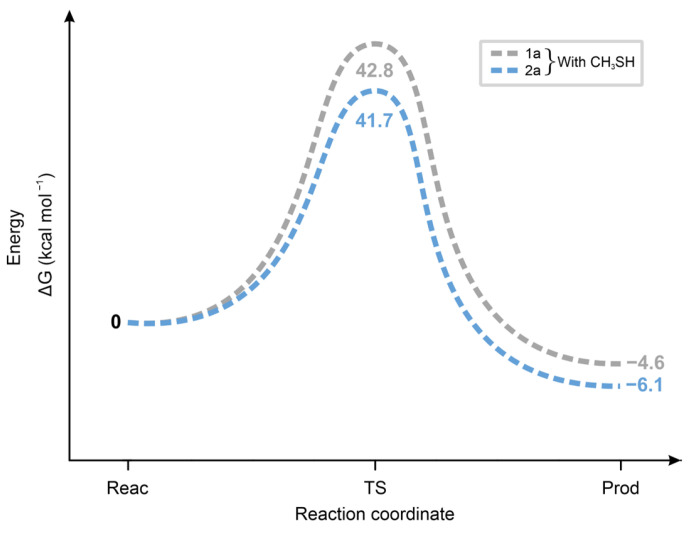
Standard free energy change (Δ*G*, kcal mol^−1^, y-axis) for the transition state formation (x-axis: Reac = reactants, TS = transition state, Prod = products) of the forward and the backward reactions of **1a** and **2a** with methanethiol (**CH_3_SH**).

As Figure 14 shows, the ΔG^o^ values of the transition states of the reactions of **1a** and **2a** with **CH_3_SH** are higher and closer to each other than those in the reactions with **CH_3_S^−^** (Figure 13). These results are in agreement with the proposed formation of a six-membered intermediate.

In comparison of the initial rate (**2b** > **2c**) of compounds **2b** and **2c** with their cancer cell cytotoxicity [25,26], it is not possible to draw a clear conclusion regarding the potential role of the investigated compounds in interacting with the reactive thiol functions of cellular proteins. Comparison of the IC_50_ values of the 4′-CH_3_ (**b**) and 4′-OCH_3_ (**c**) derivatives of the four series (**1**-**4**), we can draw a qualitative relationship between the IC_50_ values and the thiol reactivity of the analogous compounds. As shown in Figure 10a–c, **4b** and **4c**—belonging to the series with the highest cancer cell cytotoxicity power—have the lowest reactivity towards GSH under pH 8.0 conditions. On the contrary, the six-membered analogs **3b** and **3c**—belonging to the series with similar average IC_50_ values—exhibit significantly higher thiol-reactivity. Finally, the most reactive **1b** and **1c**, as well as the least reactive **2b** and **2c**, exhibit the lowest cytotoxicity. Similar conclusions can be drawn from a comparison of the NAC-reactivity of the respective compounds as well.

In addition to the reactivity with the deprotonated thiolate form, the reactions of chalcone analogs **1a** and **2a** were also evaluated using the neutral thiol, methanethiol (**CH_3_SH**), under the same conditions. This comparison aims to investigate the impact of the nucleophile’s protonation state on the reactivity profile of the compounds. Table 9 summarizes the calculated half-life values obtained for each compound at different **CH_3_SH** concentrations.

The half-life values presented in Table 9, expressed in years (yr), indicate that the reactions of both chalcone derivatives with the neutral thiol **CH_3_SH** proceed extremely slowly, in sharp contrast to the much faster reactions observed with the thiolate form (**CH_3_S^−^**). Compound **2a** exhibits shorter half-lives than **1a** at all **CH_3_SH** concentrations tested, confirming its higher intrinsic reactivity. Nonetheless, even at elevated **CH_3_SH** concentrations (e.g., 0.1 mol·L^−1^), the half-lives remain in the order of hundreds of millions of years, emphasizing the low nucleophilicity of the protonated thiol under these conditions. This data strongly supports the notion that thiolate species (RS^−^) are the primary reactive forms in biological or mildly basic environments, while the neutral thiols contribute little to the overall reaction rate due to unfavorable energetics and orbital interactions. The results reinforce the importance of the nucleophile’s ionization state in evaluating thiol-chalcone reactivity in both physiological and synthetic contexts.

## 4. Materials and Methods

### 4.1. Chemicals and Reagents

Chalcones **2a**-**c** were synthesized as reported before [25]. Their purity was tested by TLC and HPLC-UV-Vis. L-Glutathione and *N*-acetyl L-cysteine was obtained from Sigma Aldrich (Budapest, Hungary). Methanol CHROMASOLV gradient for HPLC was obtained from Honeywell (Honeywell, Hungary). Trifluoroacetic acid HiperSolve CHROMANORM was obtained from VWR (Budapest, Hungary), and formic acid from Fischer Chemicals (Budapest, Hungary). Mobile phases used for HPLC measurements were degassed in an ultrasonic water bath before use.

### 4.2. Preparation of Solutions

Solutions of glutathione (GSH) and *N*-acetylcysteine (NAC) were prepared in distilled water to a final volume of 1.5 cm^3^, yielding a concentration of 2.0 × 10^−1^ mol L^−1^ (corresponding to 0.3 mmol thiol). The pH of the solutions (pH 3.2, pH 6.3, pH 8.0) were set using 0.1 mol L^−1^ HCl and 0.1 mol L^−1^ NaOH solutions. Chalcone solutions were freshly prepared by dissolving the respective compounds in 4.6 cm^3^ of HPLC-grade methanol to obtain a final concentration of 6.5 × 10^−3^ mol L^−1^ (0.03 mmol chalcone). The GSH/NAC and chalcone solutions were preincubated in the dark at 37 °C for 15 min in a thermostated water bath. Subsequently, the solutions were mixed to afford a 10:1 molar ratio of thiol to chalcone. The resulting mixtures were maintained in the dark at 37 °C for 315 min under controlled temperature conditions. Control experiments were performed using the same protocol but missing the thiols from the aqueous solutions.

To monitor the reaction kinetics, aliquots were withdrawn at 15, 45, 75, 105, 135, 165, 195, 225, 255, 285, and 315 min, and analyzed by reversed-phase high-performance liquid chromatography (RP-HPLC).

For the determination of the initial (0 min) peak areas of compounds **2a**-**c**, methanolic solutions (4.6 cm^3^, 2.0 × 10^−1^ mol L^−1^) were prepared for each derivative. These solutions were diluted with 1.5 cm^3^ of the corresponding aqueous buffer (adjusted to the respective pH) prior to analysis. Before mixing, all solutions were preincubated at 37 °C for 15 min.

To compare the products obtained from the previously described light-induced *E*/*Z* isomerization of the parent chalcones [32] with those formed via non-photochemical (retro-Michael addition) pathways, the methanolic solutions of **2a**-**c** were exposed to diffuse laboratory light for one week. The resulting samples were analyzed by HPLC coupled with UV–Vis detection and mass spectrometry (HPLC–UV–Vis and HPLC–MS).

### 4.3. RP-HPLC-UV-Vis Measurements

Chromatographic analyses were carried out using an Agilent 1100 high-performance liquid chromatography (HPLC) system equipped with a UV–Vis detector. The detection wavelength was set to 260 nm. Separation was achieved on a Zorbax Eclipse XDB-C8 column (150 mm × 4.6 mm i.d., 5 µm particle size; Agilent Technologies, Waldbronn, Germany). The injection volume was 10 µL. Data acquisition and processing were performed using Agilent ChemStation software (version B.03.01).

Gradient elution was conducted at a flow rate of 1.2 mL min^−1^. The mobile phase consisted of solvent A (water containing 0.1% trifluoroacetic acid) and solvent B (methanol containing 0.1% trifluoroacetic acid). The gradient program was as follows: an initial isocratic period of 8 min at 40% solvent B, followed by a linear increase to 60% B over 4 min, and a subsequent linear increase to 90% B within 3 min. This was followed by a 5 min isocratic hold at 90% B. The column was then re-equilibrated to the initial conditions by a linear decrease to 40% B over 2 min, followed by a 3 min isocratic period.

### 4.4. HPLC-MS Measurements

High-performance liquid chromatography coupled with electrospray ionization mass spectrometry (HPLC–ESI–MS) analyses were carried out using an Ultimate 3000 liquid chromatography system (Dionex, Sunnyvale, CA, USA) coupled to a Thermo Q Exactive Focus quadrupole–Orbitrap hybrid mass spectrometer (Thermo Scientific, Waltham, MA, USA). Full-scan mass spectra were acquired over the *m*/*z* range of 100–1300 Da. Data acquisition was performed using Q Exactive Focus 2.1 and Xcalibur 4.2 software (Thermo Scientific).

Analyses of the compounds and their adducts were conducted in both positive and negative heated electrospray ionization (HESI) modes under the following conditions: spray voltage, 3500 V; vaporizer temperature, 300 °C; capillary temperature, 350 °C; sheath and auxiliary gas flow rates, 30 and 10 arbitrary units, respectively; and mass resolution, 35,000 at *m*/*z* 200.

Chromatographic separation was achieved on an Accucore C18 analytical column (150 mm × 2.1 mm i.d., 2.6 µm particle size) equipped with an Accucore C18 guard column (5 mm × 2.1 mm, 2.6 µm particle size). The injection volume was 5 µL, and the flow rate was maintained at 0.4 mL min^−1^. Data processing and evaluation were performed using Xcalibur 4.2 and FreeStyle 1.7 software (Thermo Scientific).

A binary gradient elution system was employed, consisting of mobile phase A (water containing 0.1% formic acid) and mobile phase B (methanol containing 0.1% formic acid). The gradient program was as follows: an initial 1 min isocratic hold at 10% B, followed by a linear increase from 10% to 95% B over 13 min, and an isocratic hold at 95% B for 3 min. The column was then re-equilibrated to 10% B within 0.1 min, followed by a 2.9 min isocratic period. The autosampler temperature was maintained at ambient temperature, and the column oven was set to 40 °C.

### 4.5. Molecular Modeling

Theoretical calculations were performed using DFT, as implemented in the G16 software package [44]. Molecular structures were optimized using the M06-2X hybrid exchange and correlation functional with long-range correction, combined with the 6-311++G(d,p) basis set [45]. The molecular coordinates were provided as part of the Supporting Information file. (Tables S2–S20). Frontier molecular orbital energies were calculated via DFT methods [45]. The electronic structure properties of the reactants (**1a**, **2a**, **3a**, **4a**, **CH_3_S^−^** and **CH_3_SH**), products, and transition states were calculated at the M06-2X/6-331G++(d,p) level using solvation model density (SMD). The SMD model has been widely used to simulate the aqueous environment in elucidating the mechanisms of organic reactions and is computationally less demanding than other continuum models [46,47,48].

The stationary points were characterized by analytic harmonic frequency calculations. The absence or presence of one imaginary frequency characterizes the optimized structures as local minima or transition states, respectively. The zero-point vibrational energy contributions were taken into account when calculating the energy barrier.

The reaction rate constant (*k*) was calculated by Equation (1) using formulations based on the thermodynamic representation of the Transition State Theory [49]:

(1) $$k\left(T\right)=\frac{k_{B}T}{h}e^{\left(\frac{-\Delta G^{‡}}{RT}\right)}$$

where *h* is the Planck’s constant, *k_B_* is the Boltzmann constant, *R* is the universal gas constant, *T* is the absolute temperature, and Δ*G*^‡^ is the activation Gibbs free energy.

From the data of the reaction rate constant of chalcone analogs (**1a**, **2a**, **3a**, and **4a**) with the methanethiol anion or methanethiol obtained via (1), it is possible to calculate the half-life time using a pseudo-first-order approximation [50] to the excess of methanethiol (Equation (2)):

(2) $$t_{1}=\ln2∕\left(k\left[x\right]\right)$$

where [*x*] is the concentration of methanethiolate anion or methanethiol in the aqueous media.

The half-life of the reaction was studied at 298 K, and [*x*] was in the range of 10^−2^–10^−1^ mol L^−1^, including the experimental concentration used in this work, 4.9 × 10^−2^ mol L^−1^.

## 5. Conclusions

This study provides a comprehensive evaluation of the thiol-reactivity of three (*E*)-2-(4-X-benzylidene)-1-indanones (**2a**-**c**), integrating kinetic analysis, stereochemical assessment, and molecular modeling. Reaction of these three compounds (**2a**-**c**) with GSH and NAC was investigated under three different pH conditions. The reactivity of the compounds showed both substituent- and pH-dependent behavior. Similarly to the open-chain (**1**), six-membered (**3**), and seven-membered (**4**) analogs, the 4-CH_3_-substituted derivative (**2b**) showed some higher reactivity than the respective OCH_3_-derivative (**2c**).

Thiol-reactivity and cancer cell cytotoxicity of **2b** and **2c** did not show a direct correlation. Comparison of thiol reactivity and cancer cell cytotoxicity of respective open chain (**1c**), six-membered (**3b**, **3c**), and seven-membered (**4b**, **4c**) analogs showed that the least reactive **4c** is the most effective, and the most reactive **1c** is one of the least effective derivatives. Based on such comparisons, lipophilicity could be one of the determining physicochemical characteristics, which increases in the order **1** < **2** < **3** < **4** [32,51]. Furthermore, three-dimensional structures have been reported to play an essential role in the four series [25,26]. Considering the present and our previous results [27,28], it is reasonable to suppose that the molecular basis of the different biological effects of **2b** and **2c** is related to noncovalent interactions of the compounds [52].

Stereochemistry of the addition of the neutral thiols onto the chalcone moiety is supposed to be determined by the conformational preference of a six-membered cyclic intermediate. Synthetic studies are necessary for a comprehensive understanding of the stereochemical course of the addition of protonated and deprotonated thiols to the investigated enone systems.

## Figures and Tables

**Figure 6:**
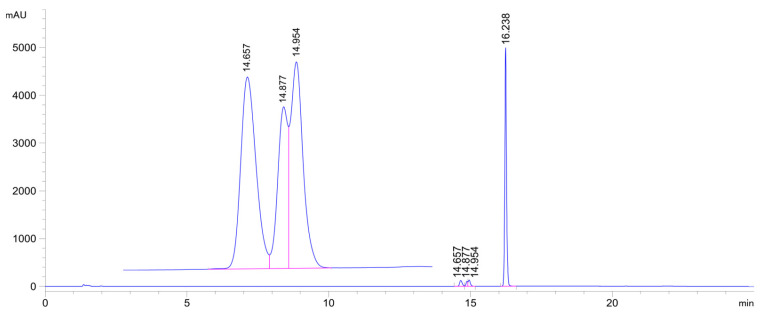
HPLC-UV chromatogram of the **2c/NAC** incubate (pH 6.3; 315 min sample). (**2c**: t_r_16.24 min, **2c-NAC-1** conjugate: t_r_14.66 min, **2c-NAC-2a** conjugate: t_r_14.88 min, **2c-NAC-2b** conjugate: t_r_14.95 min.).

**Table 1:** Retention times (t_R_) ^1^ and integrated peak areas (A) of the investigated (*E*)-2-arylidene-1-indanones (**2a**, **2b**, and **2c**) and their GSH adducts ^2^.

pH ^3^	Compound	t_R_ (*E*)-Isomer	Area Ratio ^4^ A_315_/A_0_	t_R_ (*Z*)-Isomer	t_R_ ^2^ GSH-1a	Area GSH-1a	t_R_ ^2^ GSH-1b	Area GSH-1b	t_R_ ^2^ GSH-2	Area GSH-2
3.2	**2a**	16.3	0.82	N.D. ^5^	12.6	199	12.8	342	13.2	1566
3.2	**2b**	16.6	0.78	N.D. ^5^	13.8	122	14.0	144	14.4	986
3.2	**2c**	16.3	0.92	N.D. ^5^	12.9	55	13.0	86	13.5	599
6.3	**2a**	16.3	0.53	N.D. ^5^	12.6	1449	12.8	1556	13.3	5727
6.3	**2b**	16.6	0.65	N.D. ^5^	13.8	1001	14.0	1143	14.3	4171
6.3	**2c**	16.2	0.82	N.D. ^5^	12.7	614	12.9	820	13.3	2868
8.0	**2a**	16.3	0.55	N.D. ^5^	12.7	1597	12.9	1834	13.3	5623
8.0	**2b**	16.6	0.64	N.D. ^5^	13.8	1192	14.0	1406	14.3	4073
8.0	**2c**	16.2	0.85	N.D. ^5^	12.7	743	12.9	1047	13.4	2699

^1^ Retention times in minutes; ^2^ Data refers to the average of two independent HPLC-UV measurements at the 315 min timepoint; ^3^ pH value of the aqueous thiol solution; ^4^ Ratios of peak areas measured at 0 and 315 min; ^5^ Not detectable.

**Table 2:** Retention times (t_R_) ^1^ and integrated peak areas (A) of the investigated (*E*)-3-arylidene-1-indanones (**2a**, **2b**, and **2c**) and their NAC adducts ^2^.

pH ^3^	Com-pound	t_R_ (*E*)-Isomer	Area Ratio ^4^ A_315_/A_0_	t_R_ ^2^ (*Z*)-Isomer	t_R_ NAC-1	Area ^2^ NAC-1	t_R_ NAC-2a	Area ^2^ NAC-2a	t_R_ NAC-2b	Area ^2^ NAC-2b
3.2	**2a**	16.3	0.67	N.D. ^5^	14.7	1069	14.9	119	15.0	286
3.2	**2b**	16.5	0.89	N.D. ^5^	15.3	700	15.5	87	15.5	212
3.2	**2c**	16.3	0.95	N.D. ^5^	14.7	361	14.9	47	15.0	134
6.3	**2a**	16.2	0.72	N.D. ^5^	14.7	2171	14.9	1165	15.0	1666
6.3	**2b**	16.5	0.77	N.D. ^5^	15.3	1318	15.4	751	15.5	1112
6.3	**2c**	16.2	0.92	N.D. ^5^	14.7	816	14.9	484	15.0	763
8.0	**2a**	16.2	0.59	N.D. ^5^	14.7	2849	14.9	1788	15.0	2375
8.0	**2b**	16.6	0.71	N.D. ^5^	15.3	1894	15.5	1212	15.6	1588
8.0	**2c**	16.3	0.88	N.D. ^5^	14.7	1314	14.9	775	15.0	1165

^1^ Retention times in minutes; ^2^ Data refers to the average of two independent HPLC-UV measurements at the 315 min timepoint; ^3^ pH value of the aqueous thiol solution; ^4^ Ratios of peak areas measured at 0 and 315 min; ^5^ Not detectable.

**Table 3:** A(GSH-2)/A(GSH-1) ratio of HPLC-UV areas (A) of the diastereomeric GSH-1 and GSH-2 peaks in the GSH-incubation of **2a**-**c** under different pH conditions.

Compound	pH	15 min	45 min	75 min	165 min	255 min	315 min
**2a**	8.0	2.04	1.70	1.66	1.65	1.64	1.64
	6.3	2.71	2.61	2.48	2.19	2.05	1.91
	3.2	2.45	2.56	2.72	2.89	2.92	2.90
**2b**	8.0	2.20	1.69	1.60	1.58	1.57	1.57
	6.3	3.19	2.89	2.69	2.31	2.06	1.95
	3.2	2.77	3.28	3.41	3.60	3.68	3.71
**2c**	8.0	2.19	1.63	1.54	1.51	1.51	1.51
	6.3	3.42	3.14	2.93	2.46	2.16	2.0
	3.2	2.36	3.31	3.68	4.17	4.23	4.25

**Table 4:** A(NAC-2)/A(NAC-1) ratio of HPLC-UV areas (A) of the diastereomeric NAC-1 and NAC-2 peaks in the GSH-incubation of **2a**-**c** under different pH conditions.

Compound	pH	15 min	45 min	75 min	165 min	255 min	315 min
**2a**	8.0	3.07	2.03	1.69	1.48	1.47	1.46
	6.3	1.16	1.10	1.12	1.20	1.26	1.30
	3.2	0.32	0.32	0.35	0.35	0.36	0.38
**2b**	8.0	3.68	2.72	2.21	1.65	1.51	1.48
	6.3	0.90	1.01	1.08	1.24	1.35	1.41
	3.2	0.35	0.36	0.36	0.38	0.41	0.43
**2c**	8.0	3.86	2.88	2.34	1.70	1.52	1.48
	6.3	1.44	1.44	1.44	1.48	1.51	1.53
	3.2	0.39	0.41	0.43	0.45	0.50	0.50

**Table 5:** Reactivity indices were obtained for **1a**, **2a**, **3a**, **4a**, **CH_3_SH**, and **CH_3_S^−^** at the M06-2X/6-311++G(d,p) level of theory.

Descriptor	1a eV	2a eV	3a eV	4a eV	CH_3_SH eV	CH_3_S^−^ eV
*E*_HOMO_	−7.89	−6.09	−7.76	−7.88	−7.98	−5.86
*E*_LUMO_	−1.70	−0.98	−1.41	−0.96	0.091	0.30
Δ*E*_HOMO-LUMO_	6.38	7.08	9.17	8.84	8.07	6.155

**Table 6:** Average IC_50_ (μM) values of the investigated derivatives of **2**, **3**, and **4** [25,26].

Series	P388	L1210	Molt 4/C8	CEM
**2**	37.0	174.0	255.0	63.0
**3**	17.6	94.0	96.9	68.9
**4**	12.8	103	88	56.7

**Table 7:** The HOMO-LUMO gap energies of the interaction of **1a**-**4a** with **CH_3_SH** and **CH_3_S^−^**.

Descriptor	1a eV	2a eV	3a eV	4a eV
*E*_HOMO_ (**CH_3_S^−^**)	−5.86	−5.86	−5.86	−5.86
*E*_HOMO_ (**CH_3_SH**)	−7.98	−7.98	−7.98	−7.98
*E*_LUMO_(**Chalcone**)	−1.70	−0.98	−1.41	−0.96
Δ*E*_HOMO-LUMO_(**CH_3_S^−^**)	4.16	4.87	4.45	4.90
Δ*E*_HOMO-LUMO_(**CH_3_SH**)	6.28	6.99	6.57	7.02

**Table 8:** Half-life of reactions of CH_3_S^−^ with chalcone (**1a**) and carbocyclic calcone analogs (**2a**-**4a**).

[CH_3_S^−^] (mol/L)	1a (s)	2a (s)	3a (s)	4a (s)
0.049	3.70 × 10^−4^	1.09 × 10^−5^	1.10 × 10^−2^	1.50 × 10^−1^
0.01	1.81 × 10^−3^	5.32 × 10^−5^	5.37 × 10^−2^	7.37 × 10^−1^
0.1	1.81 × 10^−4^	5.32 × 10^−6^	5.37 × 10^−3^	7.37 × 10^−2^

**Table 9:** Half-life of reactions of **CH_3_SH** with **1a** and **2a**.

[CH_3_SH] (mol/L)	1a (yr)	2a (yr)
0.049	1.60 × 10^12^	2.60 × 10^11^
0.01	7.86 × 10^12^	1.28 × 10^12^
0.1	7.86 × 10^11^	1.28 × 10^11^

## Data Availability

The original contributions presented in this study are included in the article/Supplementary Materials. Further inquiries can be directed to the corresponding author.

## Supplementary Materials

The following supporting information can be downloaded at https://www.mdpi.com/article/10.3390/ijms262110573/s1.

## References

[1] Bognár G., Kenari F., Pintér Z., Borges I.D., Camargo A.J., Oliveira H.C.B., Sanches-Neto F.O., Carvalho-Silva V.H., Napolitano H.B., Perjési P. (2024). (*E*)-2-Benzylidenecyclanones: Part XX. Reaction of Cyclic Chalcone Analogs with Cellular Thiols: Unexpected Increased Reactivity of 4-Chromanone-Compared to 1-Tetralone Analogs in Thia-Michael Reactions. Molecules.

[2] Salehi B., Quispe C., Chamkhi I., El Omari N., Balahbib A., Sharifi-Rad J., Bouyahya A., Akram M., Iqbal M., Docea A.O. (2021). Pharmacological properties of chalcones: A review of preclinical including molecular mechanisms and clinical evidence. Front. Pharmacol..

[3] Rudrapal M., Khan J., Dukhyil A.A.B., Alarousy R.M.I.I., Attah E.I., Sharma T., Khairnar S.J., Bendale A.R. (2021). Chalcone scaffolds, bioprecursors of flavonoids: Chemistry, bioactivities, and pharmacokinetics. Molecules.

[4] Rajendran G., Bhanu D., Aruchamy B., Ramani P., Pandurangan N., Bobba K.N., Oh E.J., Chung H.Y., Gangadaran P., Ahn B.C. (2022). Chalcone: A promising bioactive scaffold in medicinal chemistry. Pharmaceuticals.

[5] Shalaby M.A., Rizk S.A., Fahim A.M. (2023). Synthesis, reactions and application of chalcones: A systematic review. Org. Biomol. Chem..

[6] Michalkova R., Mirossay L., Kello M., Mojzisova G., Baloghova J., Podracka A., Mojzis J. (2023). Anticancer potential of natural chalcones: In vitro and in vivo evidence. Int. J. Mol. Sci..

[7] Wang S., Li C., Zhang L., Sun B., Cui Y., Sang F. (2023). Isolation and biological activity of natural chalcones based on antibacterial mechanism classification. Bioorg. Med. Chem..

[8] Mazumder R., Ichudaule, Ghosh A., Deb S., Ghosh R. (2024). Significance of chalcone scaffolds in medicinal chemistry. Top. Curr. Chem..

[9] Villa S.M., Heckman J., Bandyopadhyay D. (2024). Medicinally privileged natural chalcones: Abundance, mechanisms of action, and clinical trials. Int. J. Mol. Sci..

[10] Adhikari S., Nath P., Deb V.K., Das N., Banerjee A., Pathak S., Duttaroy A.K. (2025). Pharmacological potential of natural chalcones: A recent studies and future perspective. Front. Pharmacol..

[11] Folmer F., Blasius R., Morceau F., Tabudravu J., Dicato M., Jaspars M., Diederich M. (2006). Inhibition of TNFα-induced activation of nuclear factor KB by Kava (*Piper methysticum*) derivatives. Biochem. Pharmacol..

[12] Laphanuwat P., Kongpetch S., Senggunprai L., Prawan A., Kukongviriyapan V. (2022). Licochalcone A induces cholangiocarcinoma cell death via suppression of Nrf2 and NF-KB signaling pathways. Asian Pac. J. Cancer Prev..

[13] de Freitas Silva M., Pruccoli L., Morroni F., Sita G., Seghetti F., Viegas C., Tarozzi A. (2018). The Keap1/Nrf2-ARE pathway as a pharmacological target for chalcones. Molecules.

[14] Egbujor M.C., Saha S., Buttari B., Profumo E., Saso L. (2021). Activation of Nrf2 signaling pathway by natural and synthetic chalcones: A therapeutic road map for oxidative stress. Expert Rev. Clin. Pharmacol..

[15] Zhuang C., Zhang W., Sheng C., Zhang W., Xing C., Miao Z. (2017). Chalcone: A privileged structure in medicinal chemistry. Chem. Rev..

[16] Gomes M.N., Muratov E.N., Pereira M., Peixoto J.C., Rosseto L.P., Cravo P.V.L., Andrade C.H., Neves B.J. (2017). Chalcone derivatives: Promising starting points for drug design. Molecules.

[17] Ducki S. (2009). Antimitotic chalcones and related compounds as inhibitors of tubulin assembly. Anticancer. Agents Med. Chem..

[18] Prakasham A.P., Saxena A.K., Luqman S., Chanda D., Kaur T., Gupta A., Yadav D.K., Chanotiya C.S., Shanker K., Khan F. (2012). Synthesis and anticancer activity of 2-benzylidene indanones through inhibiting tubulin polymerization. Bioorg. Med. Chem..

[19] Singh A., Fatima K., Singh A., Behl A., Mintoo M.J., Hasanain M., Ashraf R., Luqman S., Shanker K., Mondhe D.M. (2015). Anticancer activity and toxicity profiles of 2-benzylidene indanone lead molecule. Eur. J. Pharm. Sci..

[20] Menezes J.C. (2017). Arylidene indanone scaffold: Medicinal chemistry and structure–activity relationship view. RSC Adv..

[21] Patil S.A., Patil R., Patil S.A. (2017). Recent developments in biological activities of indanones. Eur. J. Med. Chem..

[22] Özdemir A., Gökbulut S., Sever B., Ciftci G.A., Altintop M.D. (2018). Synthesis and evaluation of a new series of arylidene indanones as potential anticancer agents. Anti-Cancer Agents Med. Chem..

[23] Srivastava A., Fatima K., Fatima E., Singh A., Singh A., Shukla A., Luqman S., Shanker K., Chanda D., Khan F. (2020). Fluorinated benzylidene indanone exhibits antiproliferative activity through modulation of microtubule dynamics and antiangiogenic activity. Eur. J. Pharm. Sci..

[24] Lévai A. (2004). Synthesis of exocyclic α,β-unsaturated ketones. Arkivoc.

[25] Dimmock J.R., Kandepu N.M., Nazarali A.J., Kowalchuk T.P., Motaganahalli N., Quail J.W., Mykytiuk P.A., Audette G.F., Prasad L., Perjési P. (1999). Conformational and quantitative structure−activity relationship study of cytotoxic 2-arylidenebenzocycloalkanones. J. Med. Chem..

[26] Dimmock J.R., Zello G.A., Oloo E.O., Quail J.W., Kraatz H.-B., Perjési P., Aradi F., Takács-Novák K., Allen T.M., Santos C.L. (2002). Correlations between cytotoxicity and topography of some 2-arylidenebenzocycloalkanones determined by X-ray crystallography. J. Med. Chem..

[27] Kenari F., Molnár S., Perjési P. (2021). Reaction of chalcones with cellular thiols. The effect of the 4-substitution of chalcones and protonation state of the thiols on the addition process. Diastereoselective thiol addition. Molecules.

[28] Kenari F., Molnár S., Borges I.D., Napolitano H.B., Perjési P. (2023). (*E*)-2-Benzylidenecyclanones: Part XVIII. Study the possible link between glutathione reactivity and cancer cell cytotoxic effects of some cyclic chalcone analogs a comparison of the reactivity of the open-chain and the seven-membered homologs. Int. J. Mol. Sci..

[29] Caccuri A.M., Antonini G., Board P.G., Parker M.W., Nicotra M., Bello M.L., Federici G., Ricci G. (1999). Proton release on binding of glutathione to alpha, mu and delta class Glutathione Transferases. Biochem. J..

[30] Rohani N., Hao L., Alexis M., Joughin B., Krismer K., Moufarrej M., Soltis A., Lauffenburger D., Yaffe M., Burge C. (2019). Acidification of tumor at stromal boundaries drives transcriptome alterations associated with aggressive phenotypes. Cancer Res..

[31] Aldini G., Altomare A., Baron G., Vistoli G., Carini M., Borsani L., Sergio F. (2018). *N-Acetylcysteine* as an antioxidant and disulphide breaking agent: The reasons why. Free Radic. Res..

[32] Perjési P. (2015). (*E*)-2-Benzylidenebenzocyclanones: Part XIII. (*E*)/(*Z*)-Isomerization of some cyclic chalcone analogues. Effect of ring size on lipophilicity of geometric isomers. Monatsh. Chem..

[33] Wolinski K., Hinton J.F., Pulay P. (1990). Efficient Implementation of the Gauge-Independent Atomic Orbital Method for NMR Chemical Shift Calculations. J. Am. Chem. Soc..

[34] Perjési P., Perjessy A., Kolehmainen E., Ősz E., Samalikova M., Linnanto J., Virtanen E. (2004). E-2-Benzylidenebenzocycloalkanones III. Studies on transmission of substituent effects on IR carbonyl stretching frequencies and ^13^C NMR chemical shifts of *E*-2-(X-benzylidene)-1-indanones. Comparison with the IR data of *E*-2-(X-benzylidene)-1-indanones, -tetralones, and -benzosuberones. J. Mol. Struct..

[35] Pearson R.G., Songstad J. (1967). Application of the principle of hard and soft acids and bases to organic chemistry. J. Am. Chem. Soc..

[36] Allen C.F.H., Humphlett W.J. (1966). The thermal reversibility of the michael reaction V. The effect of the structure of certain thiol adducts on cleavage. Can. J. Chem..

[37] Klopman G. (1967). Chemical reactivity and the concept of charge- and frontier-controlled reactions. J. Am. Chem.Soc..

[38] Ho T.L. (1975). The hard soft acids bases (HSAB) principle and organic chemistry. Chem. Rev..

[39] Klopman G. (1983). The control of chemical reactivity. J. Mol. Struct. TEOCHEM.

[40] LoPachin R.M., Gavin T., DeCaprio A., Barber D.S. (2012). Application of the hard and soft, acids and bases (HSAB) theory to toxicant–target interactions. Chem. Res. Toxicol..

[41] Hoser A., Kaluski Z., Maluszynska H., Orlov V.D. (1980). 2-Benzylidene- l-indanone. Acta Cryst..

[42] LoPachin R.M., Gavin T., Geohagen B.C., Das S. (2007). Neurotoxic mechanisms of electrophilic type-2 alkenes: Soft–soft interactions described by quantum mechanical parameters. Toxicol. Sci..

[43] Bernáth G., Fülöp F., Kálmán A., Argay G., Sohár P., Pelcer I. (1984). Stereochemical studies-75. Saturated heterocycles-62. Connection between the diastereoselectivity and the dominant conformation in the formation of condensed-skeleton 1,3-oxazines, First X-ray diffraction evidence of N-outside conformation. Tetrahedron.

[44] Frisch M., Trucks G., Schlegel H., Scuseria G., Robb M., Cheeseman J., Scalmani G., Barone V., Petersson G., Nakatsuji H. (2016). Gaussian 16 Revision C. 01. 2016.

[45] Zhao Y., Truhlar D.G. (2008). The M06 suite of density functionals for main group thermochemistry, thermochemical kinetics, noncovalent interactions, excited states, and transition elements: Two new functionals and systematic testing of four M06-class functionals and 12 other functionals. Theor. Chem. Acc..

[46] Zhang G., Musgrave C.B. (2007). Comparison of DFT methods for molecular orbital eigenvalue calculations. J. Phys. Chem. A.

[47] Weiner P.K., Langridge R., Blaney J.M., Schaefer R., Kollman P.A. (1982). Electrostatic potential molecular surfaces. Proc. Natl. Acad. Sci. USA.

[48] Náray-Szabó G., Ferenczy G.G. (1995). Molecular electrostatics. Chem. Rev..

[49] Sanches-Neto F.O., Coutinho N.D., Aquilanti V., Silva W.A., Carvalho-Silva V.H. (2023). Mechanism and kinetics of the degradation of nitazoxanide and hydroxychloroquine drugs by hydroxyl radicals: Theoretical approach to ecotoxicity. J. Braz. Chem. Soc..

[50] Sanches-Neto F.O., Coutinho N.D., Palazzetti F., Carvalho-Silva V.H. (2020). Temperature dependence of rate constants for the H(D) + CH_4_ reaction in gas and aqueous phase: Deformed transition-state theory study including quantum tunneling and diffusion effects. Struct. Chem..

[51] Perjési P., Takács M., Ősz E., Pintér Z., Vámos J., Takács-Novák K. (2005). In-solution and on-plate light-catalyzed *E*/*Z* isomerization of cyclic chalcone analogues. Lipophilicity of *E*- and *Z*-2-(X-benzylidene)-1-benzosuberones. J. Chromatogr. Sci..

[52] Kozurkova M., Tomeckova V. (2020). Interaction of chalcone derivatives with important biomacromolecules. Chalcones and Their Synthetic Analogs.

