# Supplementary material for: (E)-2-Benzylidenecyclanones: Part XXI—Reaction of Cyclic Chalcone Analogs with Cellular Thiols: Comparison of Reactivity of (E)-2-Arylidene-1-Indanone with -1-Tetralone and -1-Benzosuberone Analogs in Thia-Michael Reactions
Source: Int J Mol Sci. 2025 Oct 30;26(21):10573. doi: 10.3390/ijms262110573 (PMC12607456; doi:10.3390/ijms262110573)
Supplement: Supplementary file 1 [file ijms-26-10573-s001.zip › ijms-3872321 Supplementary Materials.pdf]

International Journal of
Molecular Sciences

MDPI

Supplementary Materials

(*E*)-2-Benzylidenecyclanones: Part XXI. Reaction of Cyclic Chalcone Analogs with Cellular Thiols. Comparison of reactivity of (E)-2-arylidene-1-indanone with -1-tetralone and -1-benzosuberone analogs in thia-Michael Reactions

Csaba Kadlecsik [1], Gábor Bognár [1], Fatemeh Kenari [1], Zoltán Pintér [1], Júlio César de Oliveira Ribeiro [3,4], Mário G. Envall [3], Valter H. Carvalho-Silva [2,3], Hamilton B. Napolitano [2] and Pál Perjési [1,2*]

[1]Institute of Pharmaceutical Chemistry, University of Pécs, H-7624, Pécs, Hungary
[2]Group of Theoretical and Structural Chemistry of Anápolis (QTEA), State University of Goiás, Anápolis, Goiás 75132-903, Brazil
[3]Laboratory for Modeling of Physical and Chemical Transformations (MPhysChem), Theoretical and Structural Chemistry Group, Research and Graduate Center, Goiás State University, Anápolis, Goiás 75132-903, Brazil
[4]Chemistry Institute, Federal University of Goiás, Goiania, Goiás 74690-900, Brazil
*Correspondence: pal.perjesi@gytk.pte.hu; Tel.: +36-72-503-650

Abstract: *In vitro* cytotoxicity of three (*E*)-3-(4′-X-benzylidene)-1-indanones (**2a**-**c**) displayed lower cytotoxicity towards murine P388 and L1210 leukemic cells as well as human Molt 4/C8 and CEM T-lymphocytes than the respective six- (**3a**-**c**) and seven-membered (**4a**-**c**) analogs. To study whether thiol reactivity – as a possible basis of their mechanism of action – correlates with the observed cytotoxicities, kinetics of the non-enzyme catalyzed reactions with reduced glutathione (GSH) and *N*-acetylcysteine (NAC) of **2a**-**c** were investigated. Furthermore, it was also the aim of the work to compare the thiol reactivity of the open-chain chalcones (**4**) and their carbocyclic analogs (**5**-**7**) with different ring sizes (n=5-7). The reactivity of the compounds and the stereochemical outcome of the reactions were evaluated using high-pressure liquid chromatography-mass spectrometry (HPLC-MS). Molecular modeling calculations were performed to rationalize the high initial rate and low conversion of the **2a** indanone in comparison with those of the carbocyclic analog tetralone (**3a**) and benzosuberone (**4a**). Thiol reactivity and cancer cell cytotoxicity showed a dependence on both the ring size and the nature of aromatic substituents.

Keywords: chalcone; benzylideneindanones; benzylidenetetralones; benzylidenebenzosuberones; anticancer activity; glutathione; *N*-acetylcysteine; thia-Michael addition; molecular electrostatic; DFT calculations

Table S1. IC_{50} (µM) data of compounds **1c**, **2a-c**, **3a-c,** and **4a-c** against murine P388 and L1210 leukemic cells as well as human Molt 4/C8 and CEM T-lymphocytes [25,26].

**Compound**	**P388**	**L1210**	**Molt 4/C8**	**CEM**
**1c**	10.6	50.4	30.0	33.5
**2a**	>50	112	42.3	41.3
**2b**	38.6	59.1	>500	67.1
**2c**	>50	45.8	226	32.6
**3a**	30.2	121	32.4	7.42
**3b**	17.7	161	500	460
**3c**	22.1	44.0	9.41	8.84
**4a**	12.7	106.0	42.7	28.9
**4b**	11.8	25.0	21.3	11.4
**4c**	1.6	0.34	0.47	0.35

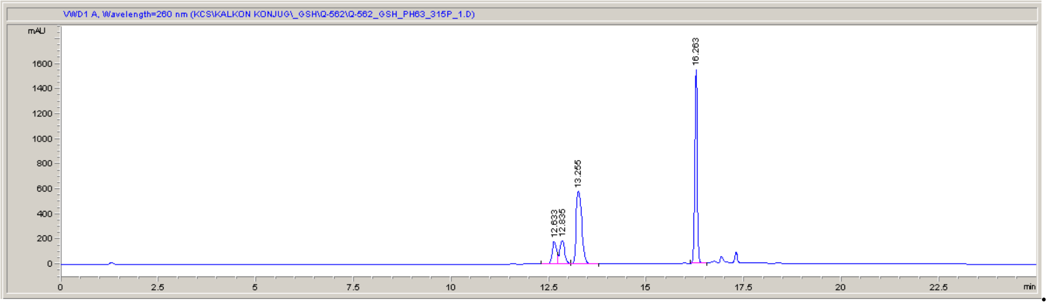

Figure S1. HPLC-UV chromatogram of the **2a/GSH** incubate (pH=6.3; 315 min sample). (**2a**: t_r16.26 min, **2a-GSH-1a** conjugate: t_r12.63 min, **2a-GSH-1b** conjugate: t_r12.84 min, **2a-GSH-2** conjugate: t_r13.26 min.).

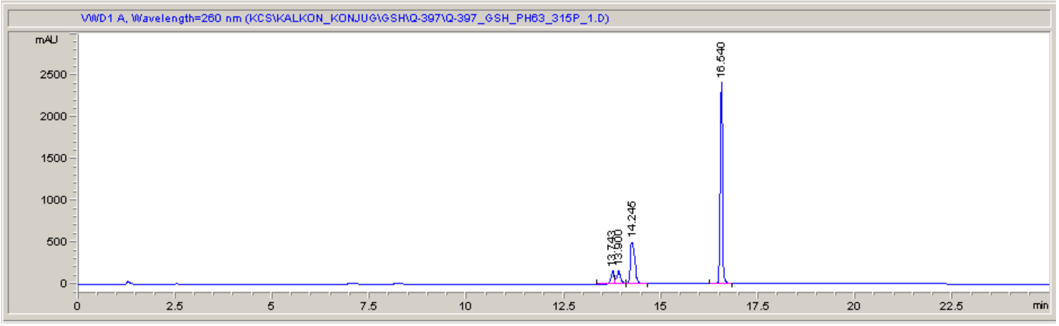

.

Figure S2. HPLC-UV chromatogram of the **2b/GSH** incubate (pH=6.3; 315 min sample). (**2b**: t_r16.54 min, **2b-GSH-1a** conjugate: t_r13.74 min, **2b-GSH-1b** conjugate: t_r13.90 min, **2a-GSH-2** conjugate: t_r14.25 min.).

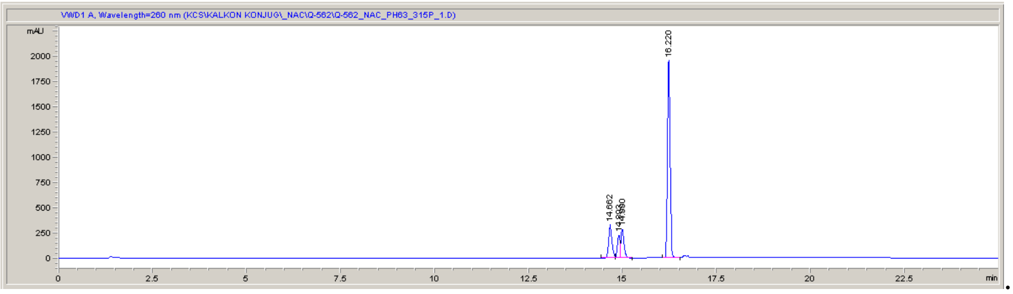

Figure S3. HPLC-UV chromatogram of the **2a/NAC** incubate (pH=6.3; 315 min sample). (**2a**: t_r16.22 min, **2a-NAC-1** conjugate: t_r14.66 min, **2a-NAC-2a** conjugate: t_r14.89 min, **2a-NAC-2b** conjugate: t_r14.99 min.).

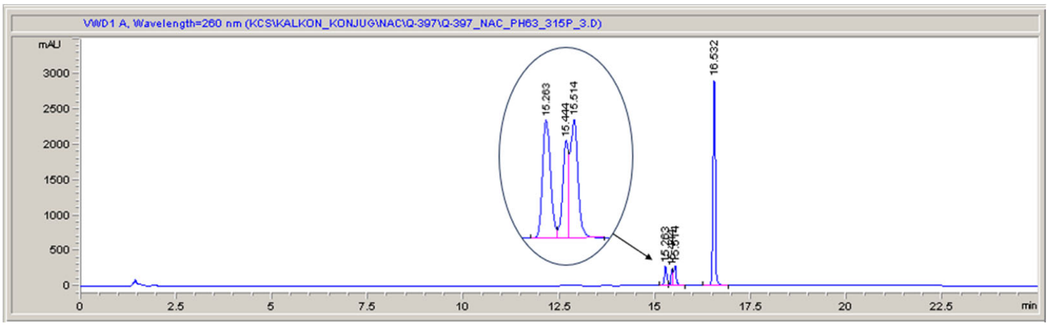

Figure S4. HPLC-UV chromatogram of the **2b/NAC** incubate (pH=6.3; 315 min sample). (**2b**: t_r16.53 min, **2b-NAC-1** conjugate: t_r15.26 min, **2b-NAC-2a** conjugate: t_r15.44 min, **2b-NAC-2b** conjugate: t_r15.51 min.).

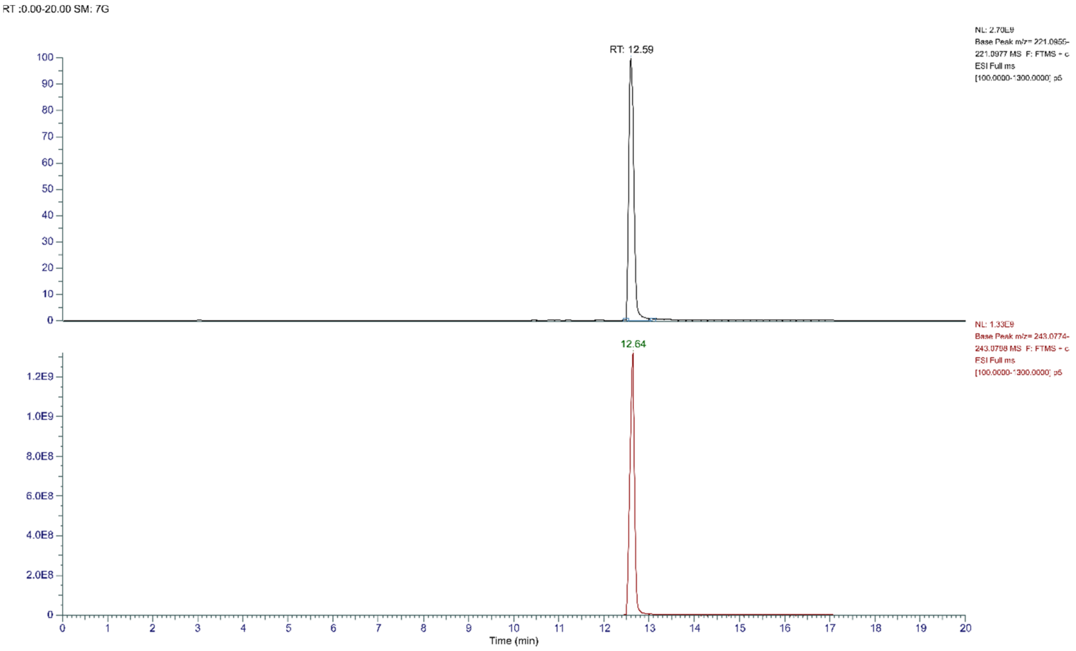

Figure S5. High resolution, positive mode HPLC-MS chromatogram of **2a** irradiated with natural light. (t_r 12.59 min: **2a** ((*E*)-**2a**), t_r 12.64 min: (*Z*)-**2a**). (Upper panel: extracted ion chromatogram of m/z 221.0966 [(**2a**)+H]$^+$. Lower panel: extracted ion chromatogram of m/z 243.0786 [(**2a**)+Na]$^+$).

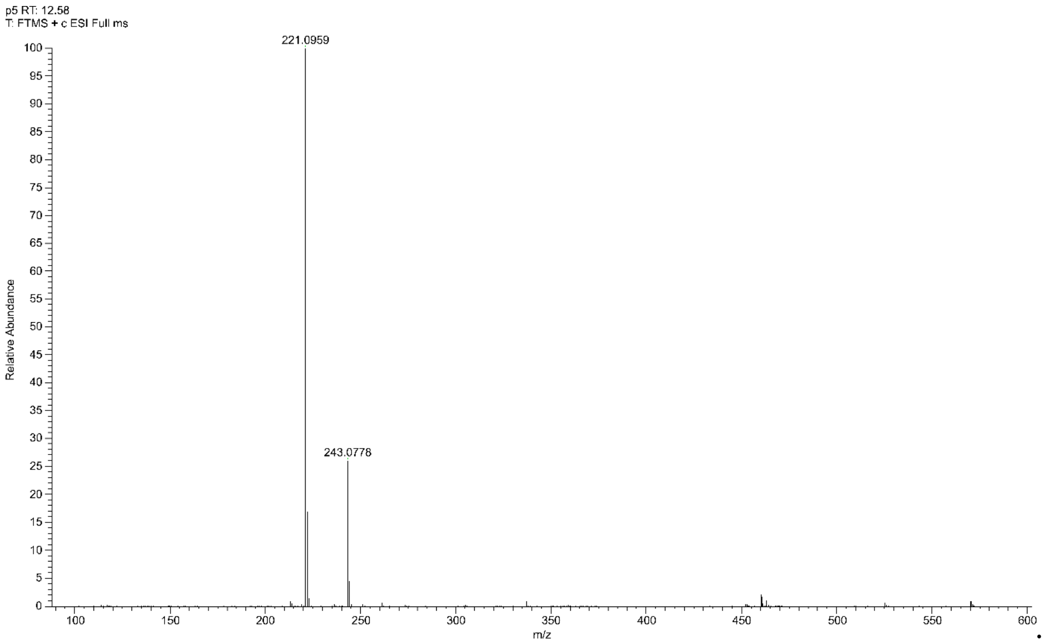

Figure S6. High resolution, positive mode HESI MS spectrum of (*E*)-**2a**. (m/z 221.0959 [(2a)+H]$^+$ and m/z 243.0778 [(**2a**)+Na]$^+$).

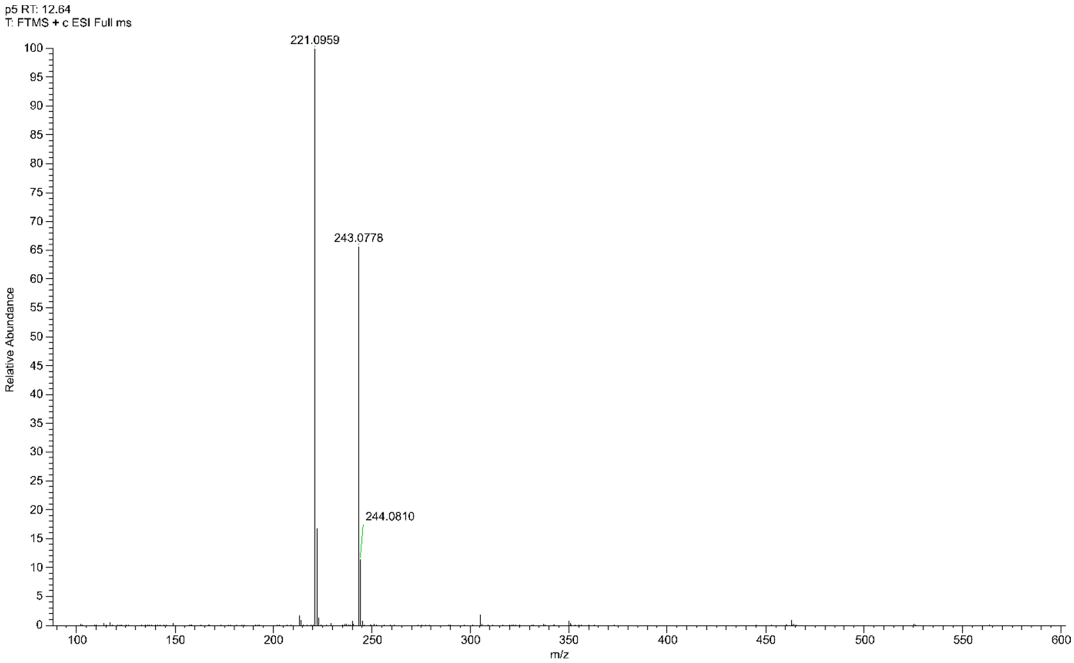

Figure S7. High resolution, positive mode HESI MS spectrum of (*Z*)-**2a.** (m/z 221.0959 [(2a)+H]$^+$ and m/z 243.0778 [(**2a**)+Na]$^+$).

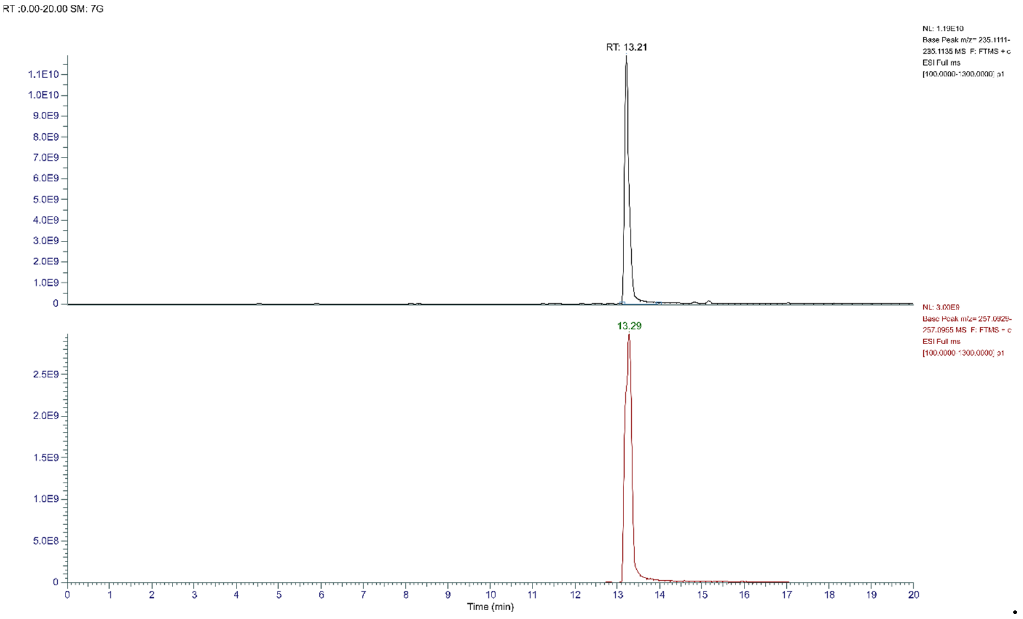

Figure S8. High resolution, positive mode HPLC-MS chromatogram of **2b** irradiated with natural light. (t_r 13.21 min: **2b** ((*E*)**-2b**), t_r 13.29 min: (*Z*)-**2b**). (Upper panel: extracted ion chromatogram of m/z 235.1123 [(**2b**)+H]$^+$. Lower panel: extracted ion chromatogram of m/z 257.0942 [(**2b**)+Na]$^+$).

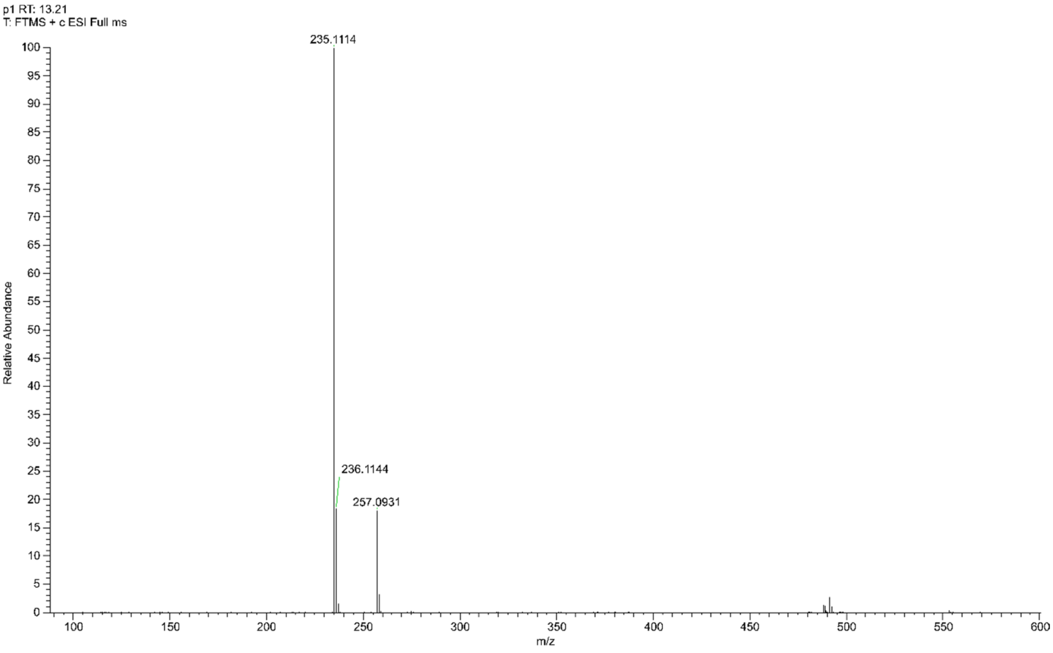

Figure S9. High resolution, positive mode HESI MS spectrum of (*E*)-**2b.** (m/z 235.1114 [(**2b**)+H]$^+$ and m/z 257.0931 [(**2b**)+Na]$^+$).

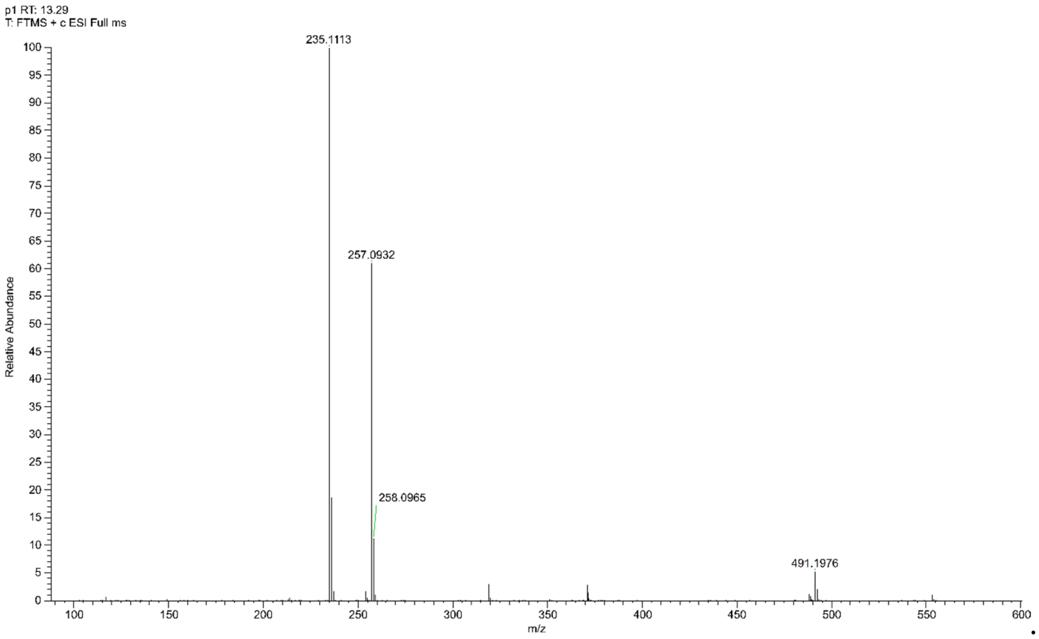

Figure S10. High resolution, positive mode HESI MS spectrum of (*Z*)-**2b.** (m/z 235.1113 [(**2b**)+H]$^+$, m/z 257.0932 [(**2b**)+Na]$^+$ and m/z 491.1976 [(**2b**)$_2$+Na]$^+$).

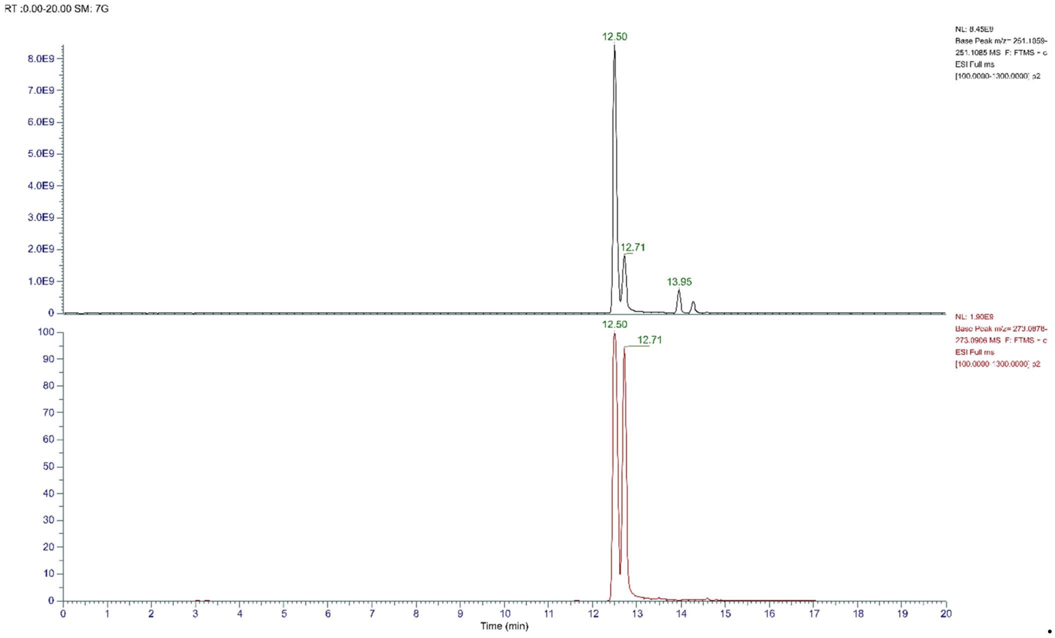

Figure S11. High resolution, positive mode HPLC-MS chromatogram of **2c** irradiated with natural light. (t_r 12.50 min: **2c** ((*E*)**-2c**), t_r 12.71 min: (*Z*)-**2c**). (Upper panel: extracted ion chromatogram of m/z 251.1072 [(**2c**)+H]$^+$. Lower panel: extracted ion chromatogram of m/z 273.0892 [(**2c**)+Na]$^+$).

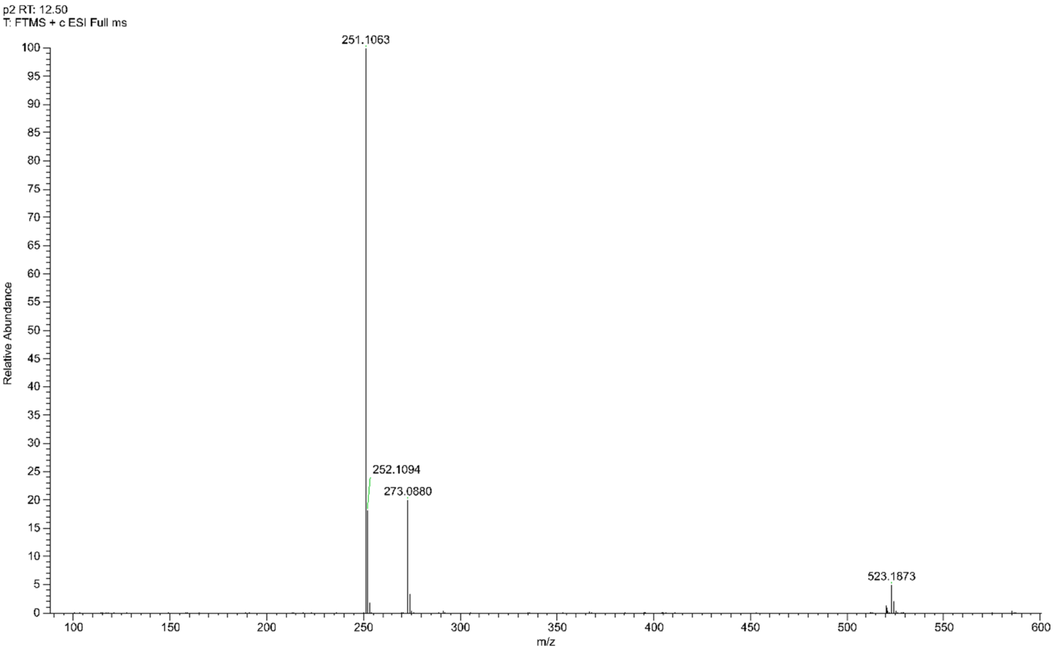

•

Figure S12. High resolution, positive mode HESI MS spectrum of (*E*)-**2c.** (m/z 251.1063 [(**2c**)+H]$^+$), m/z 273.0880 [(**2c**)+Na]$^+$ and m/z 523.1873 [(**2c**)$_2$+Na]$^+$).

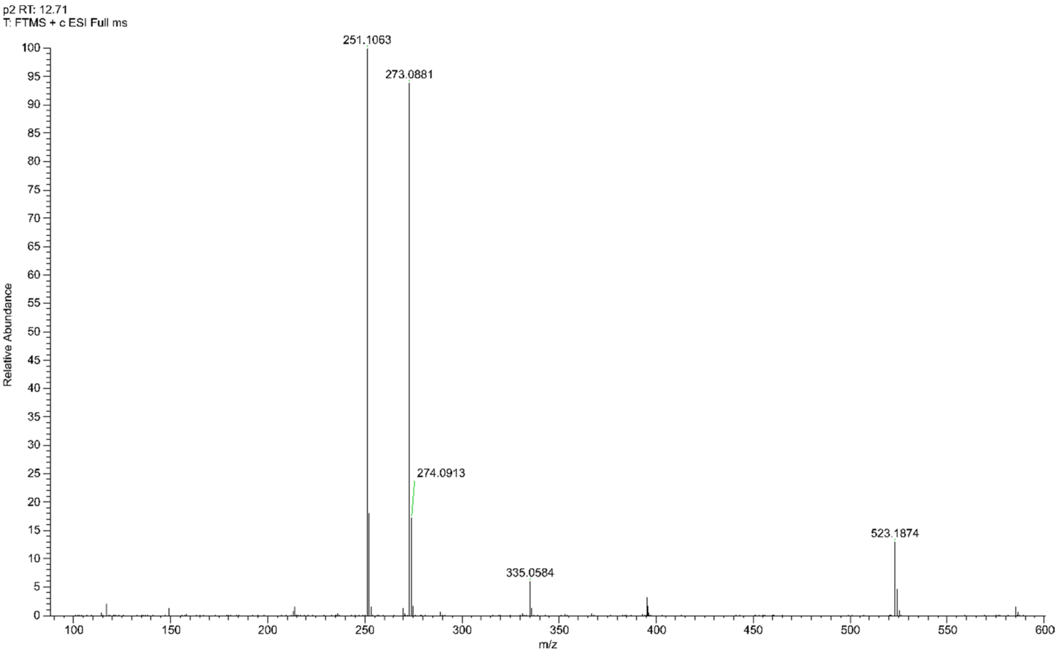

•

Figure S13. High resolution, positive mode HESI MS spectrum of (*Z*)-**2c.** (m/z 251.1063 [(**2c**)+H]$^+$), m/z 273.0881 [(**2c**)+Na]$^+$ and m/z 523.1874 [(**2c**)$_2$+Na]$^+$).

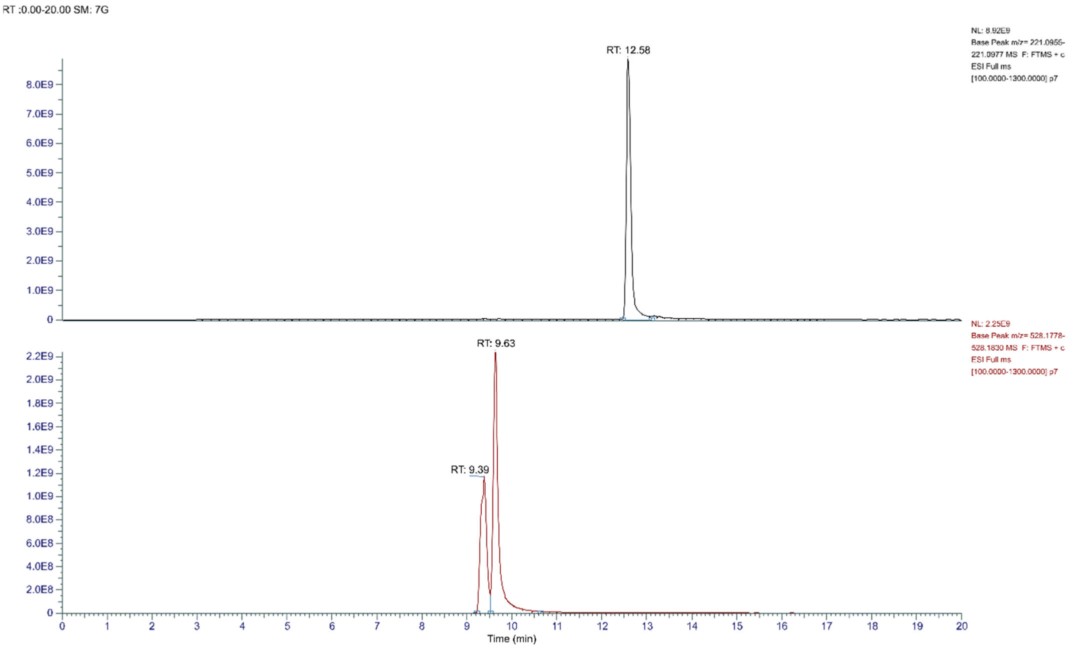

.

Figure S14. High resolution, positive mode HPLC-MS chromatograms of the **2a/GSH** incubate (pH 6.3; 315-minute sample). Upper panel: extracted ion chromatogram of m/z 221.0960 [(**2a**)+H]⁺ t_r 12.58 min: **2a**. Lower panel: extracted ion chromatogram of m/z 528.1801 [(**2a-GSH**)+H]⁺, t_r 9.39 min**:** **2a-GSH-1**, t_r 9.63 min: **2a-GSH-2**.

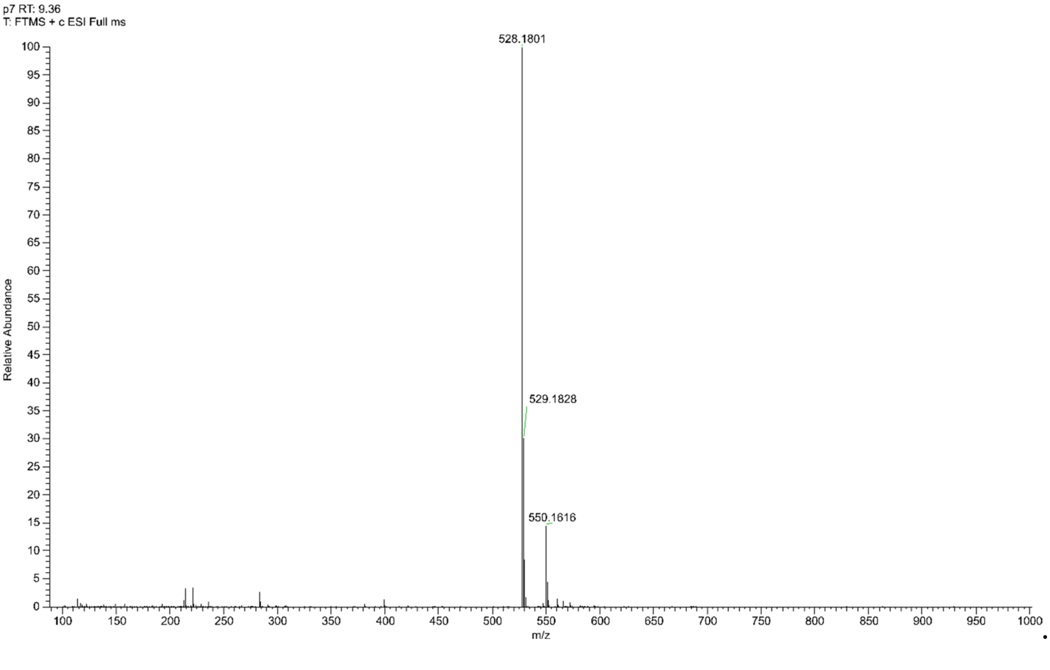

.

Figure S15. High resolution, positive mode HESI MS spectrum of the **2a-GSH-1** conjugate (t_r 9.36 min) formed in the 315-minute sample of the pH 6.3 incubate. (m/z 528.1801 [(**2a-GSH**)+H]⁺ and m/z 550.1616 [(**2a-GSH**)+Na]⁺).

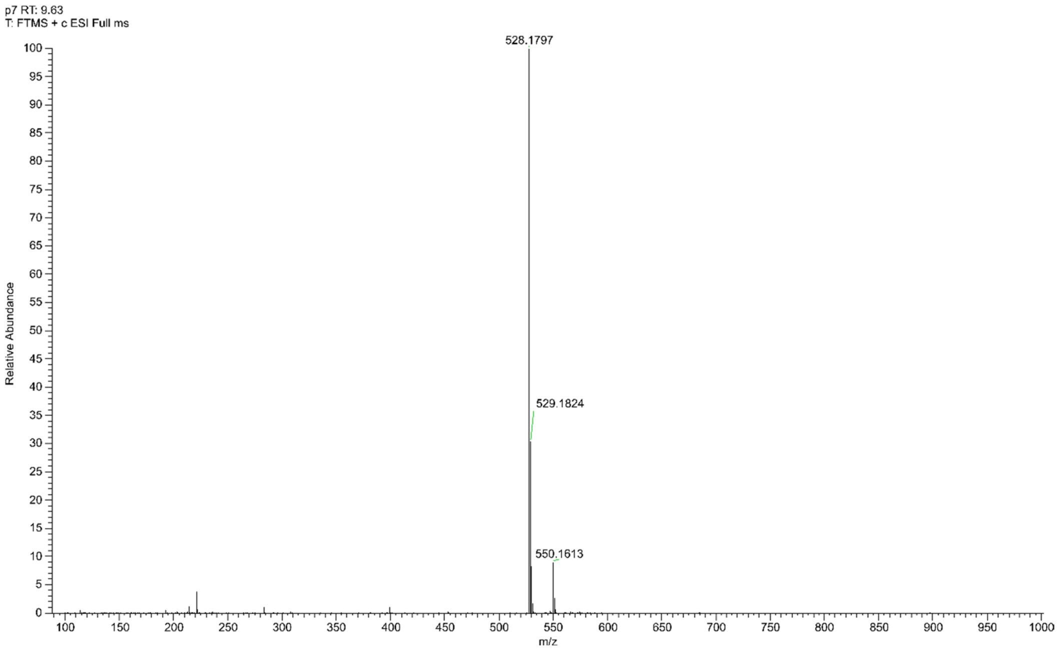

Figure S16. High resolution, positive mode HESI MS spectrum of the **2a-GSH-2** conjugate (t_r 9.63 min) formed in the 315-minute sample of the pH 6.3 incubate. (m/z 528.1797 [(**2a-GSH**)+H]$^+$ and m/z 550.1613 [(**2a-GSH**)+Na]$^+$).

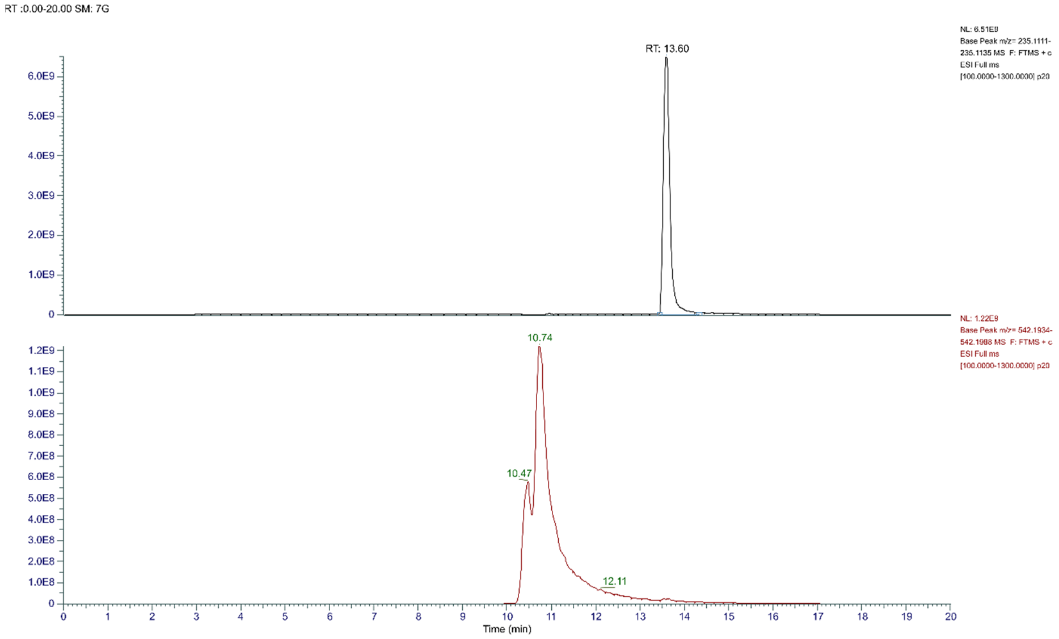

Figure S17. High resolution, positive mode HPLC-MS chromatograms of the **2b/GSH** incubate (pH 6.3; 315-minute sample). Upper panel: extracted ion chromatogram of m/z 235.1123 [(2b)+H]$^+$ t_r 13.60 min: **2b**. Lower panel: extracted ion chromatogram of m/z 542.1961 [(**2b-GSH**)+H]$^+$, t_r 10.47 min: **2b-GSH-1**, t_r 10.74 min: **2b-GSH-2**.

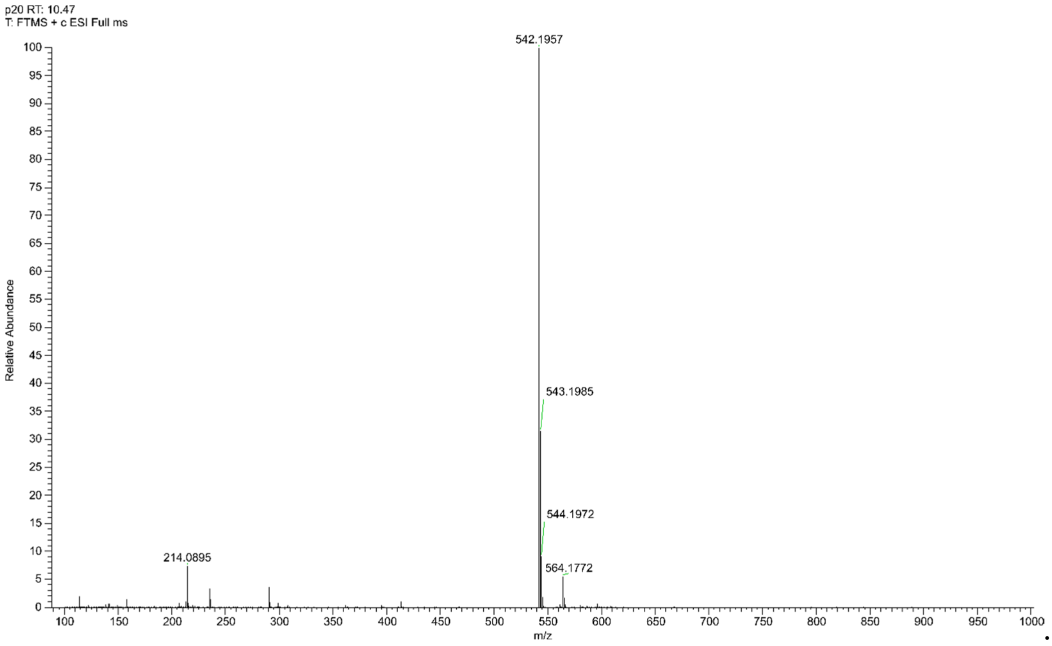

Figure S18. High resolution, positive mode HESI MS spectrum of the **2b-GSH-1** conjugate (t_r 10.47 min) formed in the 315-minute sample of the pH 6.3 incubate. (m/z 542.1957 [(**2b-GSH**)+H]$^+$ and m/z 564.1772 [(**2b-GSH**)+Na]$^+$).

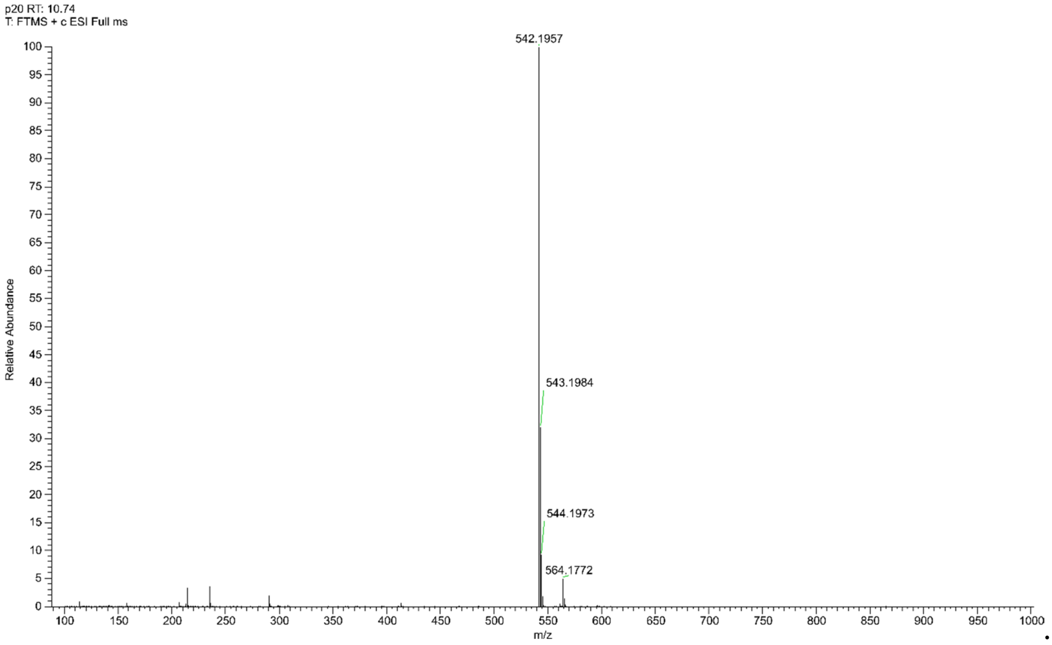

Figure S19. High resolution, positive mode HESI MS spectrum of the **2b-GSH-2** conjugate (t_r 10.74 min) formed in the 315-minute sample of the pH 6.3 incubate. (m/z 542.1957 [(**2b-GSH**)+H]$^+$ and m/z 564.1772 [(**2b-GSH**)+Na]$^+$).

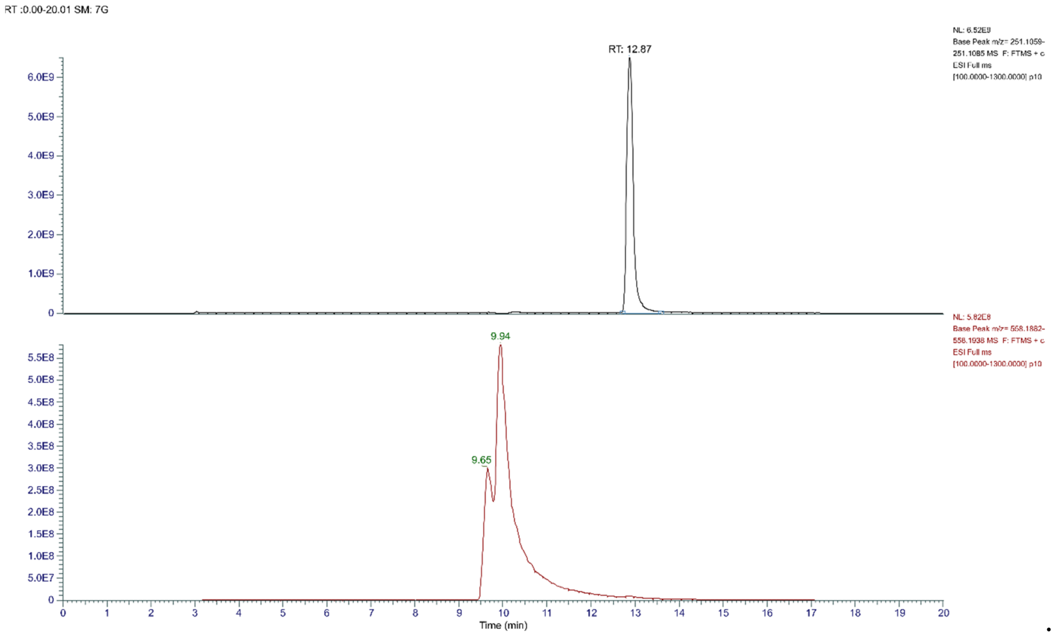

Figure S20. High resolution, positive mode HPLC-MS chromatograms of the **2c/GSH** incubate (pH 6.3; 315-minute sample). Upper panel: extracted ion chromatogram of m/z 251.1065 [(**2c**)+H]$^+$ t_r 12.87 min: **2c**. Lower panel: extracted ion chromatogram of m/z 558.1905 [(**2c-GSH**)+H]$^+$, t_r 9.65 min: **2c-GSH-1**, t_r 9.94 min: **2c-GSH-2**.

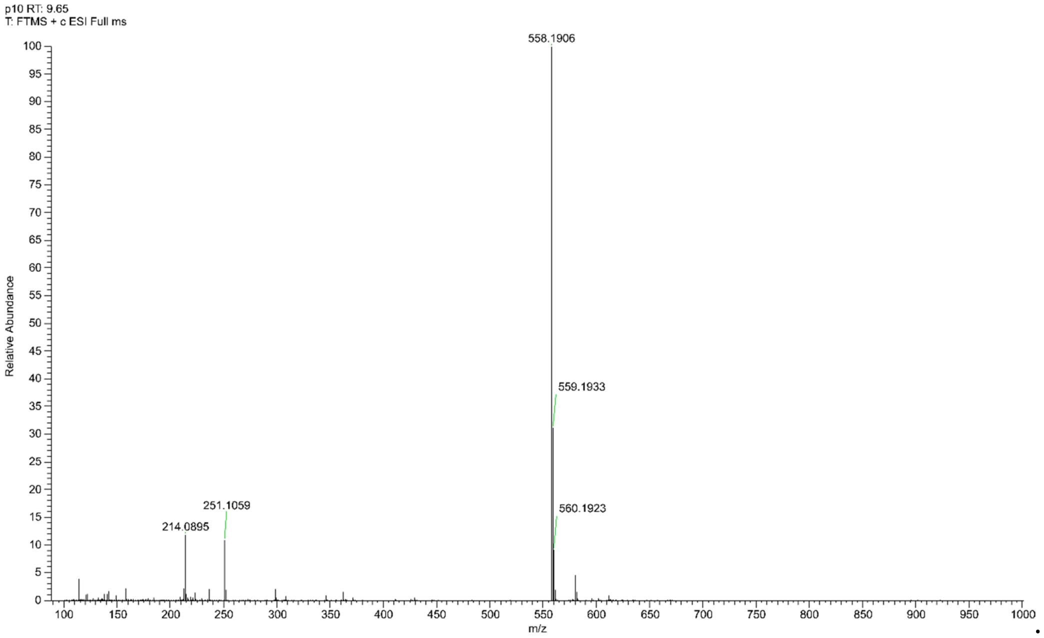

Figure S21. High resolution, positive mode HESI MS spectrum of the **2c-GSH-1** conjugate (t_r 9.65 min) formed in the 315-minute sample of the pH 6.3 incubate. (m/z 558.1906 [(**2c-GSH**)+H]$^+$).

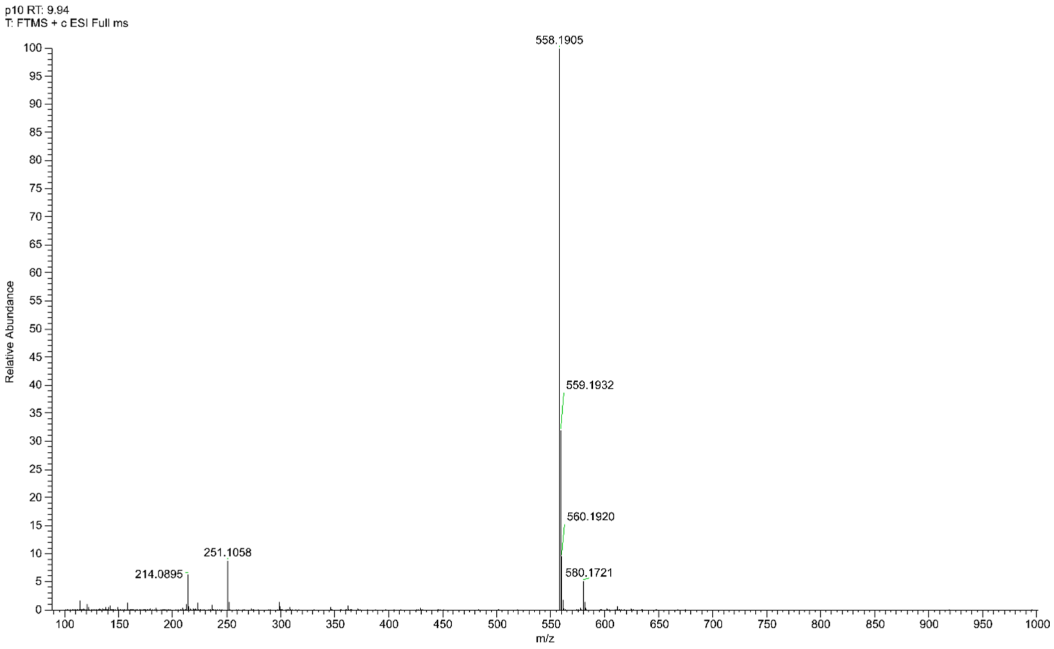

.

Figure S22. High resolution, positive mode HESI MS spectrum of the **2c-GSH-2** conjugate (t_r 9.94 min) formed in the 315-minute sample of the pH 6.3 incubate. (m/z 558.1905 [(**2c-GSH**)+H]$^+$).

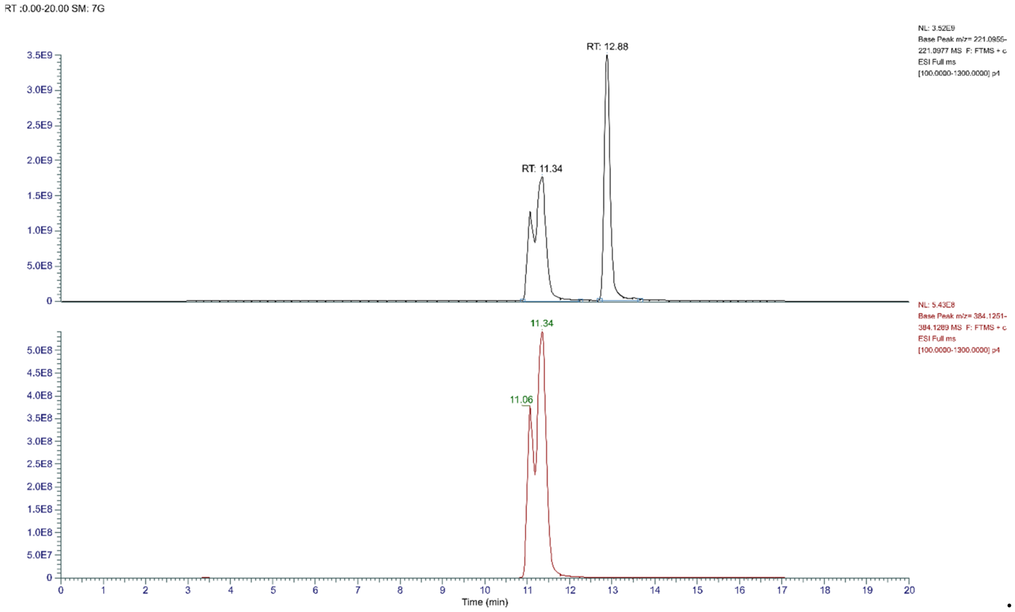

.

Figure S23. High resolution, positive mode HPLC-MS chromatograms of the **2a/NAC** incubate (pH 6.3; 315-minute sample). Upper panel: extracted ion chromatogram of m/z 221.0959 [(**2a**)+H]$^+$ t_r 12.88 min: **2a**. Lower panel: extracted ion chromatogram of m/z 384.1260 [(**2a-NAC**)+H]$^+$, t_r 11.06 min: **2a-NAC-1**, t_r 11.34 min: **2a-NAC-2**.

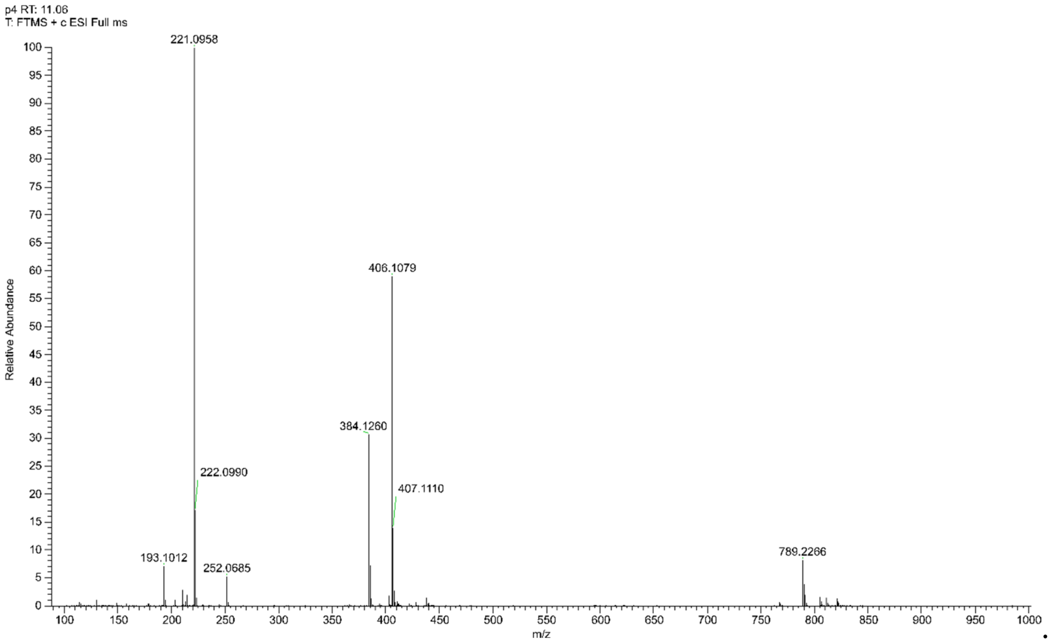

Figure S24. High resolution, positive mode HESI MS spectrum of the **2a-NAC-1** conjugate (t_r 11.06 min) formed in the 315-minute sample of the pH 6.3 incubate. (m/z 384.1260 [(**2a-NAC**)+H]$^+$, m/z 406.1079 [(**2a-NAC**)+Na]$^+$ and m/z 789.2266 [(**2a-NAC**)$_2$+Na]$^+$).

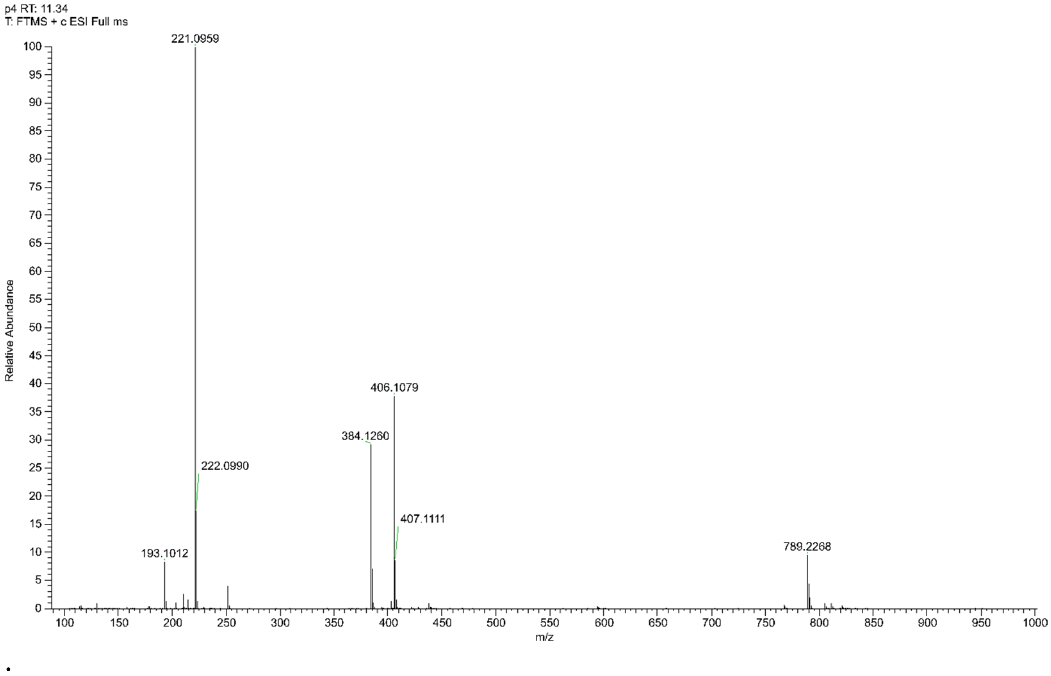

Figure S25. High resolution, positive mode HESI MS spectrum of the **2a-NAC-2** conjugate (t_r 11.34 min) formed in the 315-minute sample of the pH 6.3 incubate. (m/z 384.1260 [(**2a-NAC**)+H]$^+$, m/z 406.1079 [(**2a-NAC**)+Na]$^+$ and m/z 789.2268 [(**2a-NAC**)$_2$+Na]$^+$).

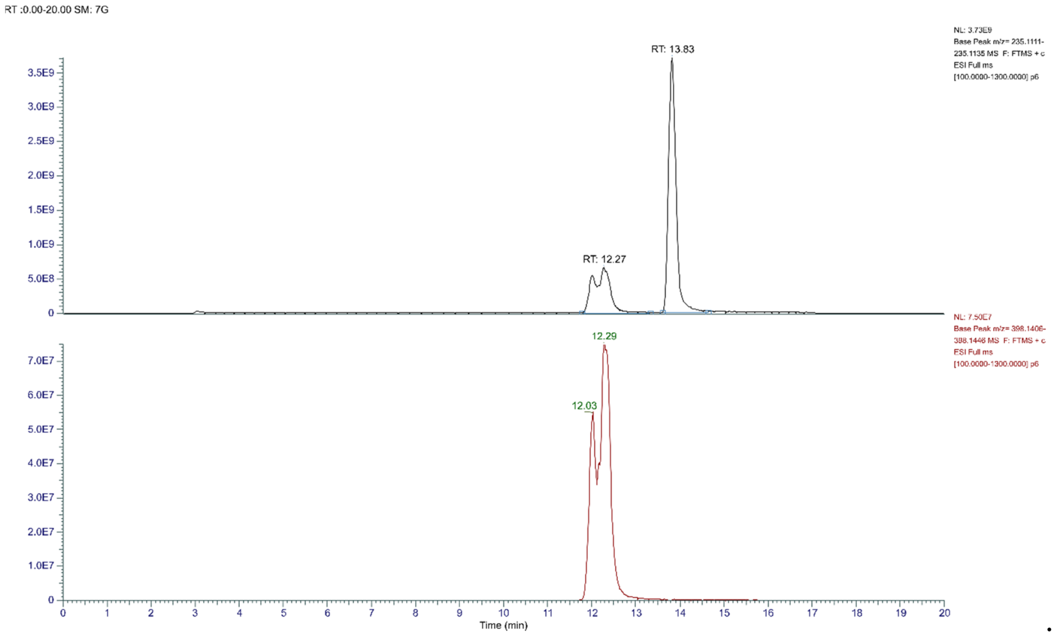

Figure S26. High resolution, positive mode HPLC-MS chromatograms of the 2b/NAC incubate (pH 6.3; 315-minute sample). Upper panel: extracted ion chromatogram of m/z 235.1123 [(**2b**)+H]$^{+}$ t_r 13.83 min: **2b**. Lower panel: extracted ion chromatogram of m/z 398.1426 [(**2b-NAC**)+H]$^{+}$, t_r 12.03 min: **2b-NAC-1**, t_r 12.29 min: **2b-NAC-2**.

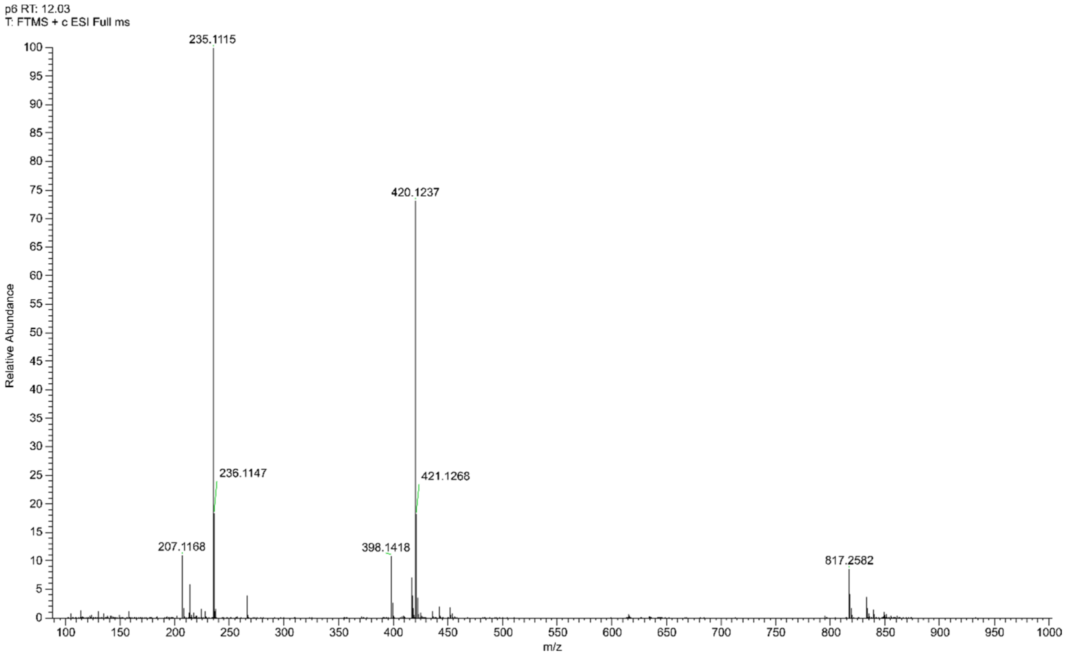

Figure S27. High resolution, positive mode HESI MS spectrum of the **2b-NAC-1** conjugate (t_r 12.03 min) formed in the 315-minute sample of the pH 6.3 incubate. (m/z 398.1418 [(**2b-NAC**)+H]$^{+}$, m/z 420.1237 [(**2b-NAC**)+Na]$^{+}$ and m/z 817.2582 [(**2b-NAC**)$_2$+Na]$^{+}$).

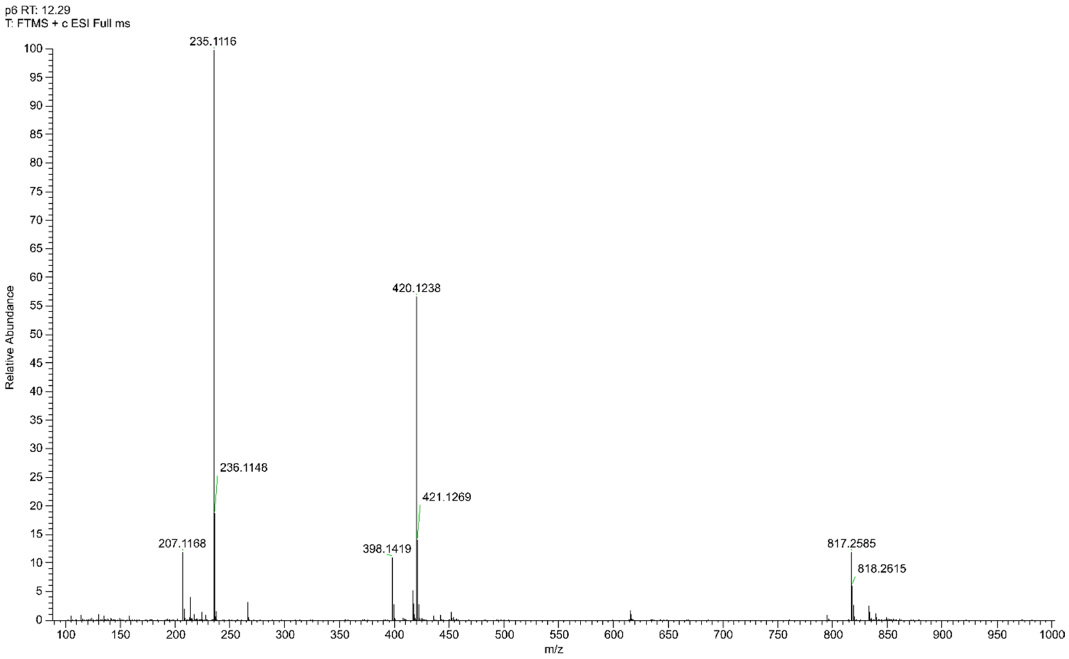

.

Figure S28. High resolution, positive mode HESI MS spectrum of the **2b-NAC-2** conjugate (t_r 12.29 min) formed in the 315-minute sample of the pH 6.3 incubate. (m/z 398.1419 [(**2b-NAC**)+H]$^+$, m/z 420.1238 [(**2b-NAC**)+Na]$^+$ and m/z 817.2585 [(**2b-NAC**)$_2$+Na]$^+$).

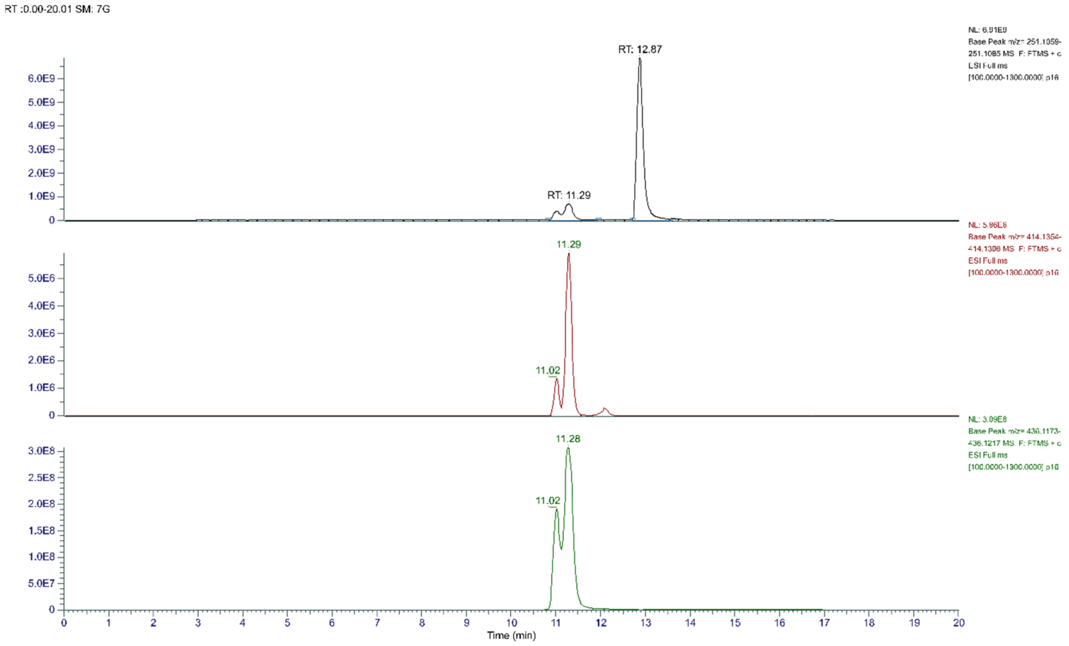

.

Figure S29. High resolution, positive mode HPLC-MS chromatograms of the **2c/NAC** incubate (pH 6.3; 315-minute sample). Upper panel: extracted ion chromatogram of m/z 251.1072 [(**2c**)+H]$^+$ t_r 12.87 min: **2c**. Middle panel: extracted ion chromatogram of m/z 414.1375 [(**2c-NAC**)+H]$^+$. Lower panel: extracted ion chromatogram of m/z 436.1188 [(**2c-NAC**)+Na]$^+$, t_r 11.02 min: **2c-NAC-1**, t_r 11.29 min: **2c-NAC-2**.

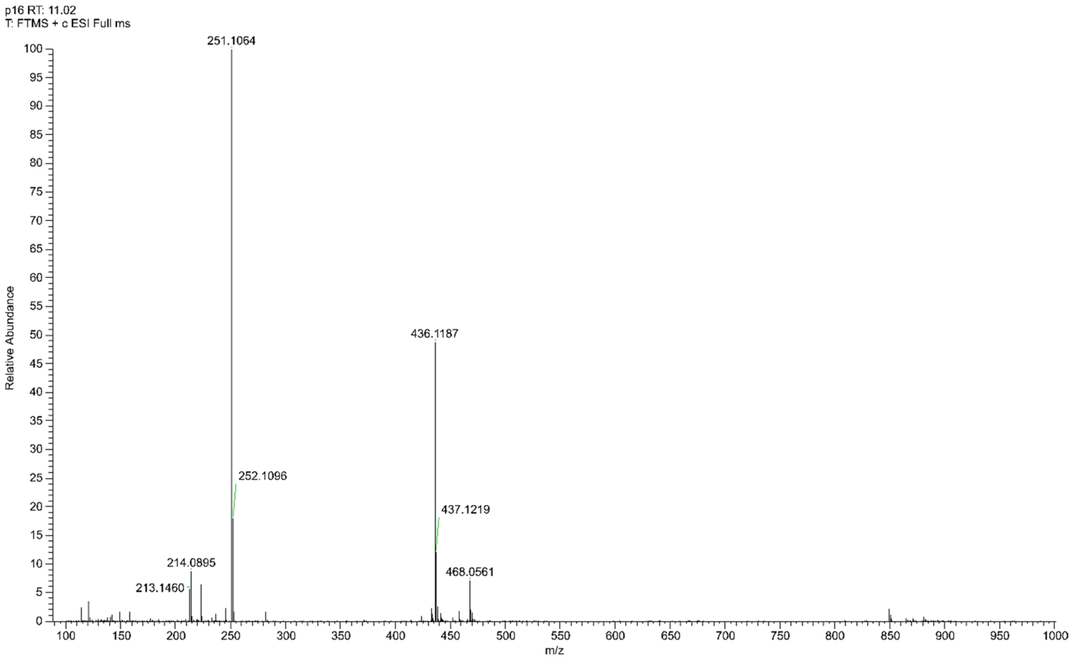

•

Figure S30. High resolution, positive mode HESI MS spectrum of the **2c-NAC-1** conjugate (t_r 11.02 min) formed in the 315-minute sample of the pH 6.3 incubate. (m/z 436.1187 [(**2c-NAC**)+Na]$^+$).

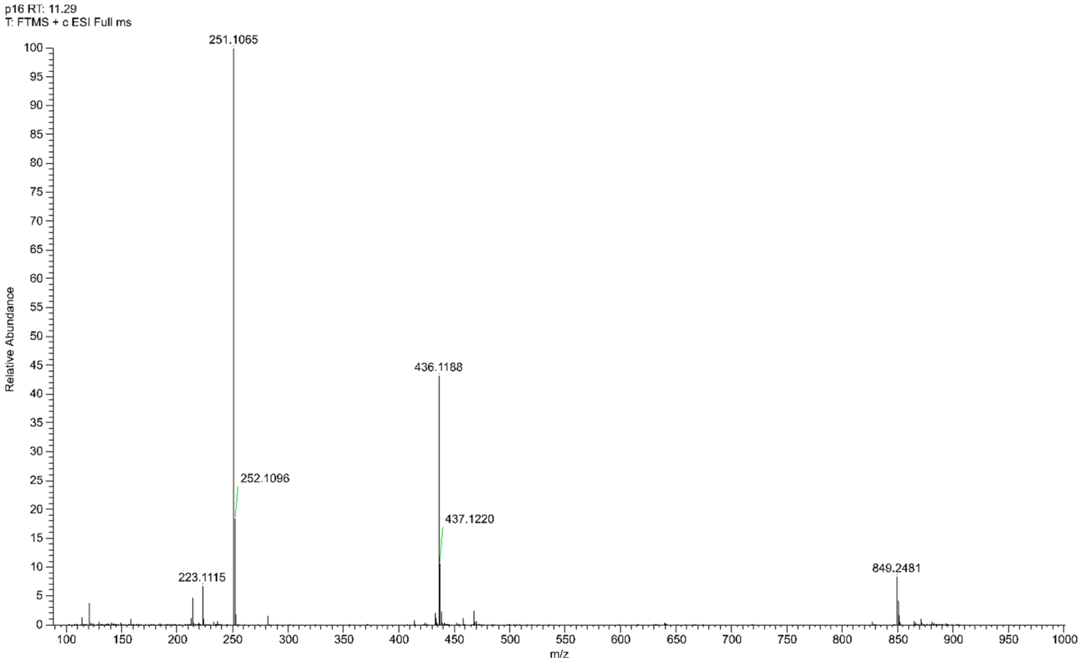

•

Figure S31. High resolution, positive mode HESI MS spectrum of the **2c-NAC-2** conjugate (t_r 11.29 min) formed in the 315-minute sample of the pH 6.3 incubate. (m/z 436.1188 [(**2c-NAC**)+Na]$^+$ and m/z 849.2481 [(**2c-NAC**)$_2$+Na]$^+$).

•

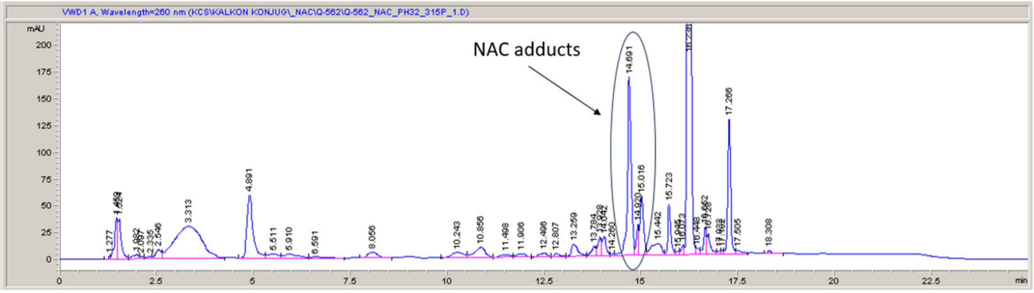

Figure S32. HPLC-UV chromatogram of NAC-incubate (pH 3.2) of **2a,** showing several peaks in the HPLC-UV chromatogram other than those of the NAC-conjugates.

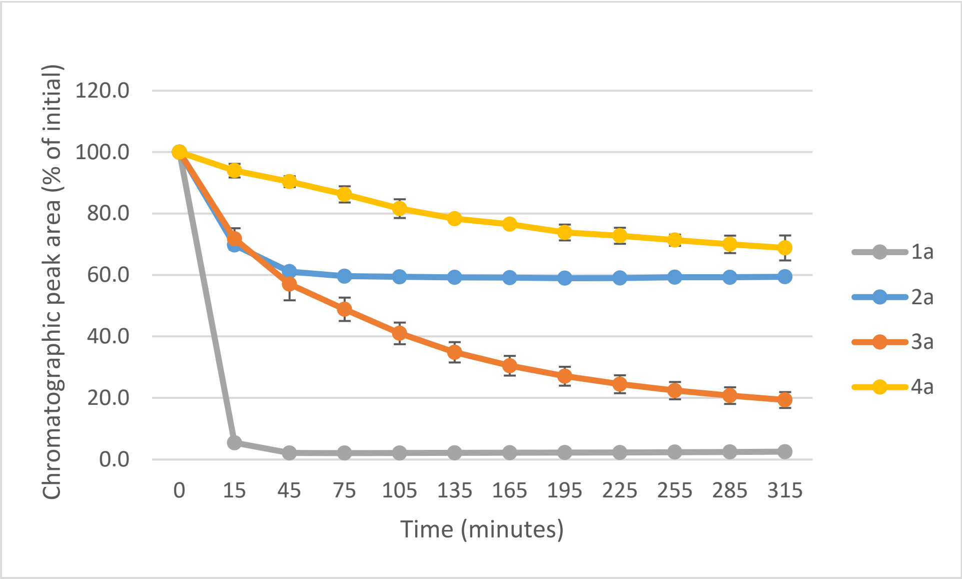

Figure S33.a. Comparison of change of HPLC-UV chromatographic peak areas of **1a** and its cyclic chalcone analogs (**2a**, **3a**, and **4a**) as a function of time (min) in the NAC incubations, pH 8.0. Each data point represents the average of two independent measurements. Error bars indicate the deviation, calculated as half the difference between two independent measurements. Due to high reproducibility, error bars may not be visually apparent at some points.

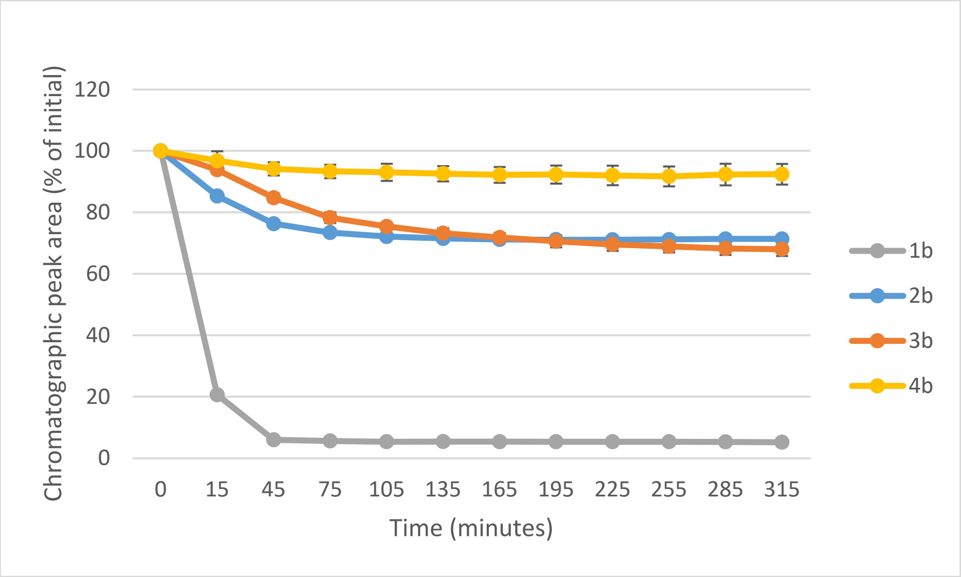

Figure S33.b. Comparison of change of HPLC-UV chromatographic peak areas of **1b** and its cyclic chalcone analogs (**2b**, **3b**, and **4b**) as a function of time (min) in the NAC incubations, pH 8.0. Each data point represents the average of two independent measurements. Error bars indicate the deviation, calculated as half the difference between two independent measurements. Due to high reproducibility, error bars may not be visually apparent at some points.

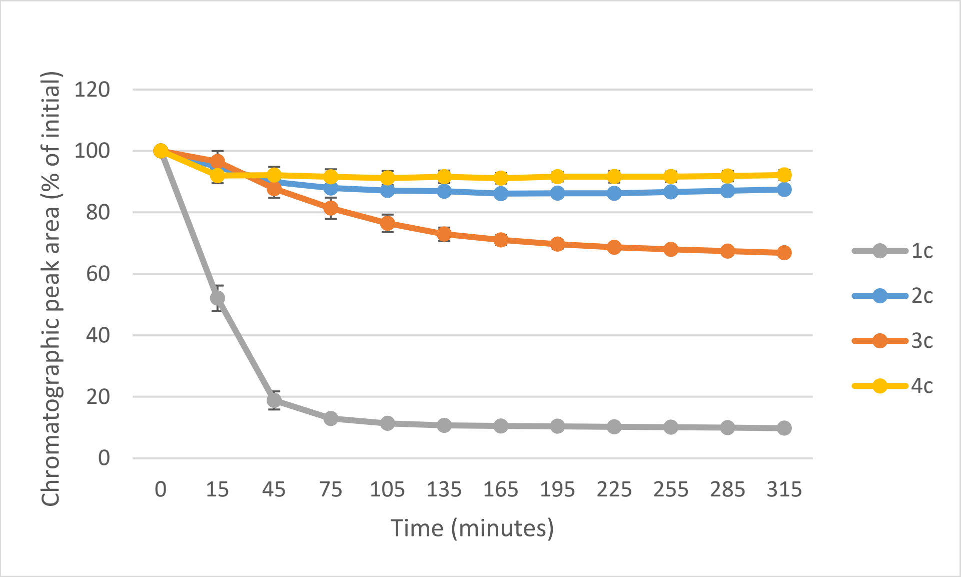

Figure S33.c. Comparison of change of HPLC-UV chromatographic peak areas of **1c** and its cyclic chalcone analogs (**2c**, **3c**, and **4c**) as a function of time (min) in the NAC incubations, pH 8.0. Each data point represents the average of two independent measurements. Error bars indicate the deviation, calculated as half the difference between two independent measurements. Due to high reproducibility, error bars may not be visually apparent at some points.

Table S2. Structural coordinates of reactant CH_3S^-

Atom	X	Y	Z
C	0.0	0.0	-1.129489
H	0.0	1.017377	-1.528469
H	0.881074	0.508689	-1.528469
H	0.881074	0.508689	-1.528469
S	0.0	0.0	0.710146

Table S3. Structural coordinates of reactant CH_3SH

Atom	X	Y	Z
C	-1.157216	0.019091	1e-06
H	-1.514979	0.526461	0.893442
H	-1.514946	0.526377	-0.893494
H	-1.526201	-1.005376	3.2e-05
S	0.662107	-0.087205	2e-06
H	0.905714	1.233263	-1.1e-05

Table S4. Structural coordinates of 1a

Atom	X	Y	Z
C	1.254676	-0.854127	0.083514
C	2.583658	-0.24266	0.012558
C	2.785243	1.108076	-0.309118
C	4.068495	1.632686	-0.357908
C	5.171664	0.82145	-0.087582
C	4.984498	-0.520241	0.229558
C	3.698601	-1.047719	0.277645
C	0.076426	-0.23417	-0.080699
H	1.243918	-1.92	0.300236
H	1.939175	1.750141	-0.524869
H	4.213006	2.677193	-0.607822

H	6.172013	1.236556	-0.126529
H	5.83727	-1.1551	0.438952
H	3.547677	-2.093521	0.52366
H	0.016254	0.826386	-0.287634
C	-1.185061	-0.998012	0.006391
O	-1.197034	-2.223247	0.059974
C	-2.473681	-0.24157	0.010851
C	-2.54345	1.098617	0.402124
C	-3.642857	-0.913752	-0.361344
C	-3.77023	1.75447	0.425177
H	-1.652569	1.630667	0.712768
C	-4.862589	-0.251616	-0.355356
H	-3.583188	-1.952365	-0.664317
C	-4.927521	1.083626	0.040971
H	-3.821085	2.789222	0.742022
H	-5.762939	-0.77334	-0.657319
H	-5.880488	1.599791	0.050358

Table S5. Structural coordinates of 2a

Atom	X	Y	Z
C	-1.100873	-1.308718	-0.157155
C	-2.364994	-0.556296	-0.096668
C	-2.568427	0.628636	-0.812978
C	-3.794699	1.280051	-0.759751
C	-4.833068	0.760507	0.011387
C	-4.647012	-0.428289	0.712321
C	-3.426662	-1.091134	0.643846
H	-1.223891	-2.389408	-0.224381
H	-1.767126	1.028463	-1.422078
H	-3.942759	2.193242	-1.324362
H	-5.786679	1.27367	0.054612

H	-5.454774	-0.844995	1.302481
H	-3.28592	-2.027509	1.173579
C	0.16147	-0.854953	-0.135305
C	1.373828	-1.755853	-0.285785
C	0.637159	0.535454	0.145212
C	2.541302	-0.817435	-0.129783
H	1.389102	-2.249984	-1.260513
H	1.390286	-2.537507	0.478651
C	2.113428	0.485015	0.119423
O	-0.030411	1.524671	0.405802
C	3.903012	-1.095775	-0.198599
C	3.010267	1.536045	0.309641
C	4.366614	1.255686	0.239005
C	4.804646	-0.052029	-0.013305
H	4.256257	-2.102685	-0.390492
H	2.649523	2.540231	0.503156
H	5.094436	2.045981	0.378104
H	5.868894	-0.252333	-0.064214

Table S6. Structural coordinates of 3a

Atom	X	Y	Z
C	-1.339838	1.495605	-0.025452
C	-2.426371	0.508688	0.135935
C	-2.223008	-0.729359	0.756421
C	-3.276296	-1.62206	0.911123
C	-4.550739	-1.290547	0.453174
C	-4.768093	-0.053008	-0.145753
C	-3.715219	0.845189	-0.291224
C	-0.063986	1.278307	-0.372491
C	0.469695	-0.052916	-0.787897
C	1.85718	-0.368947	-0.347104

C	2.377628	-1.641703	-0.614707
C	3.652308	-1.97962	-0.193278
C	4.419692	-1.038079	0.495973
C	3.910048	0.226577	0.758896
C	2.623462	0.58185	0.347373
C	2.081859	1.95762	0.646844
C	0.984031	2.359153	-0.340379
O	-0.172659	-0.829064	-1.479571
H	-1.620326	2.525545	0.189051
H	-1.23785	-0.987423	1.130312
H	-3.105401	-2.57548	1.397595
H	-5.370776	-1.988593	0.574868
H	-5.75858	0.217667	-0.492714
H	-3.887096	1.814347	-0.747868
H	1.766565	-2.358714	-1.150151
H	4.052111	-2.965353	-0.398024
H	5.421316	-1.292604	0.823342
H	4.515072	0.956974	1.285527
H	1.670445	1.961491	1.662709
H	2.90067	2.679447	0.628224
H	1.427408	2.474245	-1.335538
H	0.533619	3.310449	-0.055493

Table S7. Structural coordinates of 4a

Atom	X	Y	Z
C	-1.339354	1.423715	-0.604856
C	-2.495987	0.541786	-0.32252
C	-3.022077	0.352607	0.959094
C	-4.145247	-0.448556	1.149512
C	-4.757171	-1.071684	0.066107
C	-4.251254	-0.875368	-1.217277

C	-3.139219	-0.064027	-1.410166
C	-0.068712	1.343458	-0.18298
C	1.627944	-0.530114	0.285387
C	1.436571	-1.920715	0.223549
C	2.462659	-2.76798	-0.156264
C	3.712523	-2.228115	-0.459005
C	3.909492	-0.858835	-0.378642
C	2.882348	0.022878	-0.012605
H	-1.544277	2.204142	-1.337701
H	-2.561399	0.846131	1.804264
H	-4.543525	-0.581676	2.148813
H	-5.628691	-1.697681	0.218378
H	-4.726787	-1.348182	-2.068806
H	-2.754907	0.099085	-2.411669
H	0.458738	-2.32331	0.463186
H	2.293369	-3.836362	-0.215412
H	4.530733	-2.875288	-0.753137
H	4.888306	-0.444307	-0.598744
C	0.400135	0.23449	0.696498
C	0.983862	2.310705	-0.677486
C	3.255979	1.487368	0.089938
C	2.127466	2.48404	0.327263
H	1.394258	1.958832	-1.631524
H	3.763941	1.755145	-0.842507
H	0.491358	3.26348	-0.877309
H	4.006671	1.585167	0.88056
H	2.546486	3.488278	0.242057
H	1.742824	2.396095	1.349551
O	-0.229605	-0.137935	1.672089

Table S8. Structural coordinates of transition state of 1a-CH_3S^-

Atom	X	Y	Z
C	-0.848022	0.333056	-0.58498
C	-2.066223	-0.47962	-0.433052
C	-2.214801	-1.419014	0.595722
C	-3.384345	-2.159494	0.711022
C	-4.424716	-1.98108	-0.201985
C	-4.287254	-1.050369	-1.22754
C	-3.118489	-0.30238	-1.337208
C	0.409159	-0.111635	-0.203875
H	-0.896832	1.085385	-1.362744
H	-1.421028	-1.553831	1.322103
H	-3.489068	-2.87669	1.517226
H	-5.335868	-2.560526	-0.108914
H	-5.09117	-0.902287	-1.939479
H	-3.016908	0.433995	-2.128095
S	-1.609626	2.182516	1.027747
C	-3.42304	1.97677	1.002137
H	-3.836478	2.204456	0.017239
H	-3.706746	0.955239	1.269533
H	-3.881131	2.656212	1.724931
H	0.50954	-0.999991	0.404651
C	1.577489	0.600259	-0.586866
O	1.558817	1.664241	-1.253074
C	2.918368	0.040666	-0.19456
C	3.098512	-0.744241	0.9491
C	4.028071	0.337481	-0.992452
C	4.361882	-1.221943	1.284117
H	2.259379	-0.96676	1.597487
C	5.287088	-0.153141	-0.666658
H	3.893805	0.949138	-1.876941

C	5.457898	-0.932834	0.475286
H	4.490381	-1.817331	2.180615
H	6.135899	0.073905	-1.301349
H	6.440246	-1.309989	0.735132

Table S9. Structural coordinates of transition state of 1a-CH_3SH

Atom	X	Y	Z
C	0.965851	0.214558	0.756604
C	2.204599	-0.480916	0.496815
C	2.344382	-1.396728	-0.56085
C	3.565247	-2.013749	-0.791619
C	4.662783	-1.724729	0.019732
C	4.539155	-0.81116	1.065349
C	3.321126	-0.190154	1.30015
C	-0.323061	-0.164206	0.107813
H	0.918759	0.797357	1.67041
H	1.500466	-1.618761	-1.203618
H	3.665728	-2.720386	-1.606794
H	5.613927	-2.210112	-0.165466
H	5.392586	-0.583902	1.692873
H	3.216413	0.527206	2.107378
S	1.14748	2.149725	-0.923817
C	2.927263	2.174648	-1.25992
H	3.31071	1.155676	-1.359685
H	3.098955	2.696704	-2.204021
H	3.466867	2.698405	-0.470222
C	-1.546359	0.35877	0.796152
O	-1.467819	1.136451	1.73389
C	-2.879857	-0.072792	0.277926
C	-3.016196	-0.729781	-0.948372
C	-4.015745	0.200132	1.046203

C	-4.276911	-1.104498	-1.40045
H	-2.147403	-0.939066	-1.560743
C	-5.272028	-0.18402	0.59717
H	-3.903998	0.707461	1.996989
C	-5.4036	-0.835303	-0.628255
H	-4.379303	-1.606271	-2.355158
H	-6.148491	0.023294	1.19949
H	-6.384522	-1.132835	-0.980435
H	-0.406682	-1.221531	-0.14223
H	-0.211048	0.499112	-0.955502

Table S10. Structural coordinates of transition state of 2a-CH_3S^-

Atom	X	Y	Z
C	-1.000143	0.579214	-0.91225
C	-2.220931	-0.151305	-0.499466
C	-2.475158	-0.496473	0.832779
C	-3.635377	-1.175216	1.176696
C	-4.566623	-1.52142	0.19442
C	-4.330896	-1.171109	-1.129797
C	-3.167978	-0.481236	-1.47168
C	0.300969	0.184602	-0.643637
C	2.279821	-0.832174	0.108474
C	3.207885	-1.673329	0.715622
C	4.56063	-1.406331	0.527341
C	4.964323	-0.318697	-0.254311
C	4.027606	0.519041	-0.860304
C	2.676479	0.253162	-0.672591
H	-1.133537	1.191308	-1.798073
H	-1.762749	-0.207907	1.596508
H	-3.822917	-1.430448	2.213544
H	-5.47241	-2.051159	0.466177

H	-5.051791	-1.425488	-1.898336
H	-2.991065	-0.197851	-2.504291
H	2.880422	-2.513727	1.318565
H	5.308649	-2.042212	0.986508
H	6.022374	-0.125616	-0.391239
H	4.351613	1.358736	-1.46586
S	-1.270755	2.751007	0.389963
C	-0.047207	2.312039	1.652212
H	0.473904	1.388942	1.355857
H	0.705907	3.094911	1.765349
H	-0.514028	2.134793	2.623289
C	0.791083	-0.901184	0.163687
C	1.466197	0.976719	-1.198315
H	1.466085	1.015827	-2.292699
H	1.434217	2.01385	-0.839855
O	0.181666	-1.799736	0.781483

Table S11. Structural coordinates of transition state of 2a-CH_3SH

Atom	X	Y	Z
C	0.837816	0.392068	0.693367
C	1.976685	-0.47183	0.48588
C	2.154711	-1.216753	-0.694216
C	3.280463	-2.009908	-0.855162
C	4.247422	-2.0696	0.149449
C	4.092599	-1.32101	1.314257
C	2.969471	-0.522243	1.479326
C	-0.448947	0.276767	-0.065387
H	0.796983	0.908026	1.649142
H	1.424309	-1.152601	-1.491875
H	3.411537	-2.579424	-1.767481
H	5.124595	-2.692441	0.018566

H	4.847587	-1.359396	2.090378
H	2.842692	0.067578	2.380951
S	1.462807	2.363716	-0.830992
C	3.253607	2.173475	-1.019972
H	3.523517	1.113171	-0.989829
H	3.547559	2.565197	-1.996296
H	3.795492	2.715803	-0.24544
C	-1.589499	1.049408	0.559282
O	-1.488762	2.08539	1.192168
H	-0.134009	0.962596	-1.072179
C	-1.02226	-1.103574	-0.434129
H	-0.747682	-1.425908	-1.438551
H	-0.676526	-1.869537	0.266513
C	-2.832771	0.314136	0.265235
C	-2.513626	-0.914502	-0.307184
C	-4.149482	0.699358	0.513791
C	-3.532036	-1.793027	-0.665717
C	-5.161677	-0.179208	0.156905
H	-4.36729	1.660302	0.966683
C	-4.849905	-1.413801	-0.429248
H	-3.305999	-2.752934	-1.116263
H	-6.198248	0.085323	0.327537
H	-5.654756	-2.08689	-0.702174

Table S12. Structural coordinates of transition state of 3a-CH_3S^-

Atom	X	Y	Z
C	1.224254	0.649171	0.917438
C	2.368448	-0.183313	0.458169
C	2.592802	-0.52181	-0.88099
C	3.693082	-1.284825	-1.243059
C	4.596891	-1.726681	-0.272925

C	4.391327	-1.38723	1.058071
C	3.288492	-0.610956	1.417603
C	-0.12884	0.353475	0.838604
C	-0.702821	-0.74126	0.108981
C	-2.207342	-0.783483	0.021734
C	-2.833122	-1.967101	-0.381725
C	-4.214535	-2.030943	-0.51249
C	-4.984901	-0.899656	-0.248877
C	-4.369179	0.282687	0.149692
C	-2.983539	0.354742	0.293249
C	-2.300686	1.623951	0.727818
C	-1.075241	1.288056	1.570862
O	-0.06608	-1.676346	-0.426521
H	1.498359	1.318427	1.726311
H	1.899208	-0.174761	-1.637314
H	3.854164	-1.534785	-2.285722
H	5.455965	-2.322402	-0.559298
H	5.089506	-1.71509	1.819836
H	3.139392	-0.335808	2.456928
H	-2.224528	-2.839247	-0.589156
H	-4.690095	-2.955897	-0.81715
H	-6.063672	-0.940316	-0.348691
H	-4.96811	1.162875	0.360197
H	-1.989841	2.191394	-0.157553
H	-3.003182	2.247821	1.284891
H	-1.413567	0.832469	2.510549
H	-0.538077	2.203195	1.82607
S	1.496446	2.703575	-0.56277
C	0.35261	2.10366	-1.835221
H	-0.258452	1.286138	-1.424549
H	-0.330572	2.892135	-2.157495

H	0.882803	1.726532	-2.712981

Table S13. Structural coordinates of transition state of 4a-CH_3S^-

Atom	X	Y	Z
C	1.327393	0.59118	0.638466
C	2.283279	-0.52756	0.365269
C	2.834317	-0.827092	-0.882518
C	3.735797	-1.875742	-1.027397
C	4.105377	-2.647099	0.073916
C	3.567947	-2.355487	1.32287
C	2.669556	-1.299343	1.465884
C	-0.050469	0.535009	0.506697
C	-2.165403	-0.697496	-0.232018
C	-2.444903	-2.074857	-0.313381
C	-3.729784	-2.571086	-0.184632
C	-4.785234	-1.679604	0.007297
C	-4.523494	-0.321884	0.076038
C	-3.225238	0.203972	-0.033868
H	1.671096	1.22933	1.448884
H	2.552735	-0.229187	-1.740085
H	4.153034	-2.091643	-2.004592
H	4.808741	-3.463614	-0.042244
H	3.849185	-2.942553	2.189812
H	2.2602	-1.07081	2.444925
H	-1.61866	-2.758467	-0.470002
H	-3.909744	-3.638552	-0.234164
H	-5.802056	-2.042527	0.104125
H	-5.345465	0.373083	0.218946
S	2.349988	2.387006	-0.849819
C	1.620879	3.745677	0.131047
H	0.550898	3.851924	-0.059345

H	1.764669	3.573306	1.201425
H	2.103698	4.692285	-0.120918
C	-0.696174	-0.368652	-0.402225
C	-0.891967	1.580024	1.203723
C	-3.137168	1.717995	0.033265
C	-1.758068	2.349097	0.195539
H	-1.545092	1.132593	1.964299
H	-3.756842	2.023076	0.882722
H	-0.221489	2.261857	1.728852
H	-3.63047	2.12524	-0.855524
H	-1.900056	3.385043	0.515639
H	-1.234454	2.386322	-0.765333
O	-0.096199	-0.98139	-1.309267

Table S14. Structural coordinates of product of 1a-CH_3S^-

Atom	X	Y	Z
C	1.04617	-0.676387	-0.205664
C	2.135268	0.369739	-0.217504
C	2.318165	1.241234	0.861405
C	3.333659	2.189939	0.842308
C	4.190903	2.280045	-0.255052
C	4.02172	1.41404	-1.329682
C	2.999945	0.464944	-1.308176
C	-0.319107	-0.138793	0.072733
H	1.049778	-1.197587	-1.165256
H	1.665528	1.167877	1.725304
H	3.460975	2.859573	1.685477
H	4.984702	3.017984	-0.2673
H	4.684713	1.472164	-2.185539
H	2.873186	-0.212692	-2.146669
S	1.443757	-1.989044	1.056739

C	3.083752	-2.509835	0.478672
H	3.041209	-2.793996	-0.574199
H	3.819317	-1.716772	0.61874
H	3.37856	-3.378862	1.06778
H	-0.405568	0.685024	0.769481
C	-1.436965	-0.663899	-0.519849
O	-1.441624	-1.632064	-1.385951
C	-2.779464	-0.05687	-0.185179
C	-3.048074	0.556751	1.044743
C	-3.809689	-0.122685	-1.129154
C	-4.298998	1.104064	1.312005
H	-2.280994	0.591859	1.809704
C	-5.060253	0.430155	-0.867023
H	-3.621907	-0.60779	-2.079734
C	-5.310834	1.047315	0.355538
H	-4.487474	1.567026	2.274206
H	-5.840706	0.376351	-1.617898
H	-6.285628	1.472514	0.564964

Table S15. Structural coordinates of product of 1a-CH_3SH

Atom	X	Y	Z
C	1.071642	-0.656345	-0.421416
C	2.33984	0.162481	-0.384124
C	2.454404	1.316654	0.393091
C	3.649172	2.033913	0.424652
C	4.743922	1.606256	-0.319222
C	4.638866	0.453783	-1.09672
C	3.446888	-0.260526	-1.125624
C	-0.196984	0.152849	-0.186687
H	1.006873	-1.144297	-1.394983
H	1.61183	1.663752	0.981045

H	3.720917	2.928435	1.032676
H	5.671484	2.166318	-0.296649
H	5.484643	0.114861	-1.68396
H	3.366956	-1.158604	-1.730265
S	1.174626	-2.089714	0.738537
C	1.658828	-1.259317	2.275293
H	0.957934	-0.464503	2.532901
H	1.626496	-2.019674	3.056133
H	2.670538	-0.860455	2.205946
C	-1.45141	-0.555492	-0.647842
O	-1.400549	-1.579272	-1.305593
C	-2.773819	0.053607	-0.301274
C	-2.872352	1.310324	0.303073
C	-3.939243	-0.657079	-0.606515
C	-4.121754	1.84869	0.593368
H	-1.98299	1.880466	0.542543
C	-5.184611	-0.121869	-0.308086
H	-3.859715	-1.630988	-1.074267
C	-5.27686	1.133412	0.291393
H	-4.192236	2.825533	1.056492
H	-6.083439	-0.679907	-0.541966
H	-6.249169	1.552978	0.522454
H	-0.1491	1.097039	-0.744831
H	-0.309837	0.430597	0.86471

Table S16. Structural coordinates of product of 2a-CH_3S^-

Atom	X	Y	Z
C	-1.048212	0.889101	-0.818144
C	-2.138682	-0.126254	-0.488647
C	-3.249857	0.140643	0.308572
C	-4.210491	-0.843622	0.547733

C	-4.081093	-2.106563	-0.016529
C	-2.979213	-2.380891	-0.827044
C	-2.020429	-1.402214	-1.054876
C	0.336115	0.415183	-0.522125
C	2.230029	-0.520613	0.402438
C	3.121429	-1.22086	1.208954
C	4.475368	-1.217224	0.866878
C	4.922193	-0.523106	-0.258577
C	4.021658	0.182548	-1.0644
C	2.677373	0.178949	-0.724973
H	-1.116047	1.081017	-1.895298
H	-3.376316	1.123117	0.746894
H	-5.06293	-0.614655	1.177509
H	-4.82811	-2.869699	0.168669
H	-2.867148	-3.359698	-1.279797
H	-1.160022	-1.62501	-1.67797
H	2.770739	-1.758274	2.084187
H	5.188665	-1.757141	1.479829
H	5.976833	-0.530702	-0.509734
H	4.374467	0.722005	-1.937714
S	-1.353872	2.59247	-0.144841
C	-0.898987	2.353974	1.59256
H	0.139501	2.030368	1.665864
H	-1.01257	3.319675	2.086005
H	-1.549097	1.624513	2.076538
C	0.752259	-0.356392	0.539274
C	1.497054	0.836249	-1.390964
H	1.389928	0.501199	-2.430784
H	1.622236	1.926428	-1.426693
O	0.091591	-0.885764	1.523648

Table S17. Structural coordinates of product of 2a-CH_3SH

Atom	X	Y	Z
C	0.911251	0.714583	0.289548
C	1.964172	-0.359969	0.131043
C	2.380749	-0.781215	-1.134923
C	3.357914	-1.760968	-1.265716
C	3.936731	-2.331013	-0.131534
C	3.529151	-1.915009	1.131106
C	2.546693	-0.933827	1.259672
C	-0.376194	0.348188	-0.464728
H	0.67842	0.820115	1.353079
H	1.942943	-0.335351	-2.022472
H	3.671038	-2.080577	-2.25311
H	4.700197	-3.093286	-0.235088
H	3.974177	-2.350964	2.018199
H	2.230525	-0.608275	2.245605
C	-1.588122	1.233854	-0.164873
O	-1.595014	2.446718	-0.084301
C	-0.860096	-1.090985	-0.173827
H	-0.555051	-1.796433	-0.947978
H	-0.448194	-1.448925	0.774973
C	-2.763999	0.354218	-0.024384
C	-2.35755	-0.975933	-0.061527
C	-4.102132	0.720263	0.120186
C	-3.307188	-1.989829	0.035575
C	-5.045342	-0.291606	0.217859
H	-4.388511	1.765861	0.147001
C	-4.645489	-1.634845	0.173499
H	-3.012829	-3.032881	0.006941
H	-6.095821	-0.04952	0.325144
H	-5.39751	-2.412169	0.249366

H	-0.205727	0.453749	-1.543963
S	1.557409	2.337833	-0.298453
C	3.145247	2.3631	0.576922
H	3.827629	1.607581	0.187692
H	3.572812	3.352679	0.413989
H	2.99271	2.21528	1.646883

Table S18. Structural coordinates of product of 3a-CH_3S^-

Atom	X	Y	Z
C	-1.30505	0.979159	-0.689908
C	-2.247141	-0.196196	-0.462989
C	-3.334149	-0.181175	0.408359
C	-4.164214	-1.296166	0.534035
C	-3.925595	-2.440847	-0.21709
C	-2.846477	-2.462587	-1.101042
C	-2.018179	-1.35367	-1.217735
C	0.161358	0.682939	-0.531213
C	0.66712	-0.17275	0.412981
C	2.139805	-0.479309	0.327905
C	2.65196	-1.629124	0.934737
C	4.005944	-1.944779	0.846153
C	4.870734	-1.102203	0.154209
C	4.371568	0.054158	-0.444486
C	3.018151	0.372956	-0.365013
C	2.453004	1.631057	-0.96732
C	1.064358	1.368157	-1.536119
O	-0.005018	-0.792014	1.344262
H	-1.452871	1.275415	-1.73546
H	-3.543665	0.705841	0.993899
H	-5.001128	-1.263447	1.222794
H	-4.572167	-3.305364	-0.120724

H	-2.651442	-3.345517	-1.699524
H	-1.17573	-1.378784	-1.90242
H	1.977241	-2.281643	1.475995
H	4.38327	-2.846379	1.315705
H	5.925789	-1.340521	0.080663
H	5.041158	0.7202	-0.980038
H	2.368345	2.388339	-0.17632
H	3.131332	2.025291	-1.727564
H	1.150946	0.769582	-2.456599
H	0.61317	2.320718	-1.831616
S	-1.801096	2.551323	0.161365
C	-1.229127	2.246194	1.852112
H	-0.167261	1.998047	1.843348
H	-1.378393	3.172622	2.407682
H	-1.79533	1.44567	2.32878

Table S19. Structural coordinates of product of 4a-CH_3S^-

Atom	X	Y	Z
C	1.482274	0.907049	0.375999
C	2.337868	-0.3511	0.306924
C	3.265518	-0.628176	-0.697054
C	4.016768	-1.80283	-0.673781
C	3.858199	-2.720329	0.358985
C	2.938819	-2.451916	1.371891
C	2.189442	-1.281111	1.342244
C	-0.003736	0.657362	0.375285
C	-2.024966	-0.651615	-0.330478
C	-2.244074	-2.031643	-0.525146
C	-3.490604	-2.61842	-0.395982
C	-4.588695	-1.819682	-0.077738
C	-4.394651	-0.462649	0.11284

C	-3.134014	0.151577	2e-06
H	1.730319	1.377341	1.332857
H	3.404587	0.064057	-1.519161
H	4.725556	-1.998515	-1.47062
H	4.443126	-3.632587	0.377022
H	2.807237	-3.154174	2.187508
H	1.473293	-1.079877	2.132959
H	-1.391551	-2.649747	-0.778442
H	-3.607213	-3.686574	-0.540606
H	-5.578347	-2.250329	0.023857
H	-5.246318	0.164835	0.359696
S	2.078116	2.186265	-0.852745
C	1.5013	3.680996	-0.003032
H	0.415036	3.754234	0.000105
H	1.880136	3.703915	1.019961
H	1.909347	4.531859	-0.549455
C	-0.574273	-0.228556	-0.501019
C	-0.845148	1.54412	1.267697
C	-3.158119	1.655964	0.224831
C	-1.837302	2.387065	0.451074
H	-1.412162	0.967677	2.012863
H	-3.793795	1.818667	1.100761
H	-0.181862	2.200118	1.836506
H	-3.692817	2.115301	-0.613759
H	-2.061132	3.333265	0.954445
H	-1.374969	2.636657	-0.50712
O	0.066755	-0.851624	-1.45209

Table S20. Energies in kcal/mol of reactants, transition states and products

Reactant **CH_3S^-**	-274975
Reactant **CH_3SH**	-275274
Reactant **1a**	-410350
Reactant **2a**	-434624
Reactant **3a**	-458929
Reactant **4a**	-483582
Transition State **1a-CH_3S^-**	-685339
Transition State **1a-CH_3SH**	-685594
Transition State **2a-CH_3S^-**	-709254
Transition State **2a-CH_3SH**	-709508
Transition State **3a-CH_3S^-**	-733917
Transition State **4a-CH_3S^-**	-758567
Product **1a-CH_3S^-**	-733902
Product **1a-CH_3SH**	-685645
Product **2a-CH_3S^-**	-709240
Product **2a-CH_3SH**	-709559
Product **3a-CH_3S^-**	-685325
Product **4a-CH_3S^-**	-758554
